# Charge-mediated remote epitaxy of Ga_2_O_3_ via MoS_2_

**DOI:** 10.1126/sciadv.aee9510

**Published:** 2026-10-16

**Authors:** Gyeong Ryul Lee, Dong-Gwan Kim, Wonwoo Suh, Yoonho Choi, Donghan Kim, Mohammad M. Afandi, Juhui Oh, Hongsik Park, Celesta S. Chang, Hyun S. Kum, Taehoon Lee, Roy B. Chung

**Affiliations:** ^1^ School of Materials Science and Engineering, Kyungpook National University, Daegu, Republic of Korea 41566.; ^2^ Department of Physics and Astronomy, Seoul National University, Seoul, Republic of Korea 08826.; ^3^ School of Electronic and Electrical Engineering, Kyungpook National University, Daegu, Republic of Korea 41566.; ^4^ KNU Advanced Material Research Institute, Kyungpook National University, Daegu, Republic of Korea 41566.; ^5^ Department of Electrical and Electronic Engineering, Yonsei University, Seoul, Republic of Korea 03722.

## Abstract

Remote epitaxy of the corundum phase α-Ga_2_O_3_ is demonstrated through a domain-free, transfer-free monolayer of MoS_2_ template synthesized on sapphire. Despite the intrinsic nonidealities of MoS_2_—specifically its thickness and polar bonding characteristics—for remote epitaxy, single-crystalline α-Ga_2_O_3_ films are epitaxially grown on polycrystalline MoS_2_ with crystallographic fidelity comparable to those grown directly on bare sapphire. This observation substantiates that the substrate lattice potential can still be effectively transmitted through the MoS_2_ layer, facilitating epitaxial registry at the growth front. The resulting α-Ga_2_O_3_ films exhibit crystal quality and surface morphology on par with those grown directly on bare sapphire. Moreover, a centimeter-scale, crack-free α-Ga_2_O_3_ membrane was successfully exfoliated using a Ni stressor, underscoring the mechanical compatibility and scalability of the remote epitaxial process. Structural disordering observed with more-than-bilayer MoS_2_ further corroborates the remote epitaxy mechanism. First-principles calculations reveal that interfacial interactions between the bottom S atoms of MoS_2_ and the Al atoms of sapphire induce spatially modulated charge distributions on the MoS_2_ surface, creating energetically favorable adsorption sites for Ga species. Collectively, these findings establish a mechanistic framework for charge-mediated remote epitaxy across polar interfaces and expand the design space for heterogeneous integration of transferable wide bandgap Ga_2_O_3_ membranes in next-generation power electronics and optoelectronic devices.

## INTRODUCTION

Since the inception in 2017, remote epitaxy has emerged as a transformative growth technique, rapidly expanding from initial demonstrations on III-V arsenide and nitride materials to encompass complex oxides and other materials (*1*–*3*). This advancement has enabled novel device architectures through heterogeneous integration, showcasing the technique’s versatility and potential (*3*–*6*).

Remote epitaxy employs a 2-dimensional (2D) van der Waals (vdW) material—most commonly graphene—as an interlayer between a single-crystalline substrate and the epitaxial layer grown atop it. The term “remote” reflects the underlying conceptual framework, which integrates lattice transparency, electrostatic potential penetration through atomically thin 2D materials, and vdW bonding at the interface. Together, these effects enable epitaxial registry without the formation of strong covalent bonds. This framework distinguishes remote epitaxy from both conventional and vdW epitaxy by balancing substrate influence and interlayer decoupling, thereby facilitating exfoliation and substrate reuse (*7*).

To date, mechanistic studies of remote epitaxy have focused predominantly on graphene as it is regarded as an ideal 2D material for this growth technique (*8*, *9*). However, from the perspectives of material compatibility and process scalability, it is essential to investigate alternative vdW materials to enable broader adoption and practical implementation of this novel epitaxial technique. Recently, the remote epitaxy of ZnO nanowires on MoS_2_-covered ZnO nanowires was reported, demonstrating the feasibility of non-graphene-based remote epitaxy (*10*). Notably, the interaction between zinc and sulfur resulted in polarity inversion in the ZnO nanowires with respect to the underling ZnO nanowires, illustrating how a 2D material can influence the growth based on the nature of its constituent atoms (*11*). Liu *et al.* has demonstrated remote epitaxy of VO_2_ using an ultrathin amorphous Al_2_O_3_ deposited by atomic layer deposition, suggesting that remote epitaxy can be facilitated by an atomically thin layer of non-vdW materials as well (*12*).

As remote epitaxy continues to evolve in sophistication and versatility, there will be more materials that can be fabricated as single-crystalline membranes, offering a high degree of integration flexibility. This is particularly important for materials like gallium oxide (Ga_2_O_3_), a wide bandgap semiconductor considered vital for next-generation high-power electronics. Ga_2_O_3_ suffers from inherently low thermal conductivity, which significantly limits its performance. To enable efficient heat dissipation, Ga_2_O_3_ typically needs to be transferred onto a thermally conductive substrate via direct wafer bonding followed by substrate removal (*13*), or through complex ion-cut techniques (*14*). Remote epitaxy and exfoliation of Ga_2_O_3_ not only enable more efficient thermal management but also expand its applicability as part of the broader family of wide bandgap semiconductors compatible with heterogenous integration (*4*).

In this work, we demonstrate centimeter-scale remote epitaxy of corundum α-Ga_2_O_3_—a metastable polymorph of Ga_2_O_3_—on MoS_2_-coated sapphire (α-Al_2_O_3_) and elucidate the remote epitaxy mechanism using 2D materials beyond graphene. Because metastable α-Ga_2_O_3_ can be grown and stabilized on sapphire (α-Al_2_O_3_) due to their shared corundum structure (*15*), its remote epitaxy would not be feasible without the substrate acting as a seed layer. This makes the α-Ga_2_O_3_/sapphire system an ideal platform for investigating MoS_2_-based remote epitaxy. From a practical perspective, α-Ga_2_O_3_ is also a promising wide-bandgap semiconductor for next-generation high-power electronic devices, alongside monoclinic β-Ga_2_O_3_ (*16*–*18*). In this study, polycrystalline MoS_2_ was directly synthesized on sapphire using a scalable solution-based process, followed by Ga_2_O_3_ growth via a simple mist chemical vapor deposition (CVD) under atmospheric pressure. Notably, no sophisticated high-vacuum epitaxy or deposition techniques were required, underscoring the robustness and processability of MoS_2_-based remote epitaxy. The overall process flow for the MoS_2_-based remote epitaxy of Ga_2_O_3_ is depicted in Fig. 1.

**Fig. 1.**
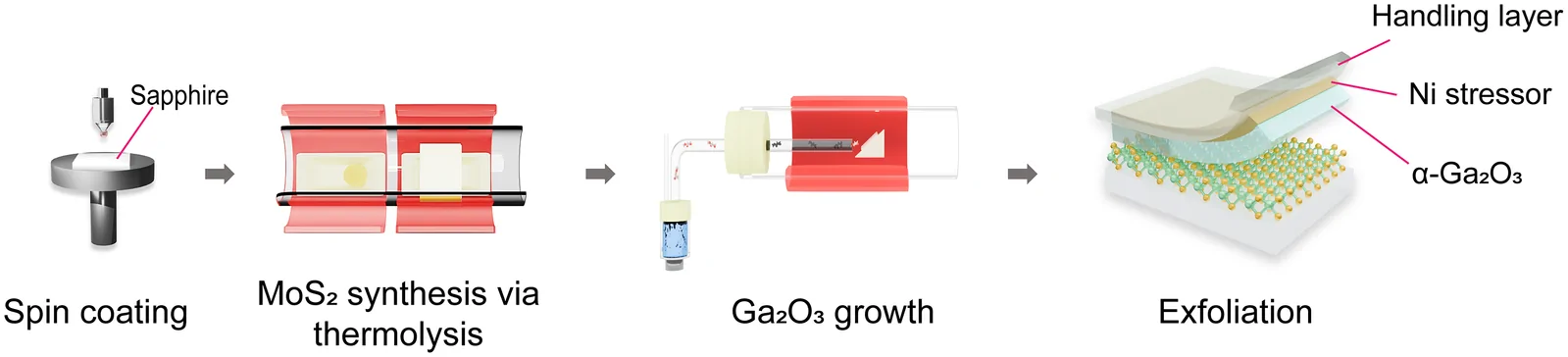
Schematic illustration of the overall process flow for MoS_2_-based remote epitaxy of Ga_2_O_3_. The process includes spin-coating of an (NH_4_)_2_MoS_4_ dissolved in solvent mixture of dimethylformamide, n-butylamine and ethanolamine, followed by sulfurization to form the MoS_2_ template. Subsequently, α-Ga_2_O_3_ is grown via mist-CVD and exfoliated using a sputtered Ni stressor layer.

## RESULTS AND DISCUSSION

### Continuous and uniform polycrystalline MoS_2_ for remote epitaxy

For remote epitaxy, it is critical that the 2D material is continuous and exhibits uniform thickness (or a well-controlled number of layers). The most extensively studied method for synthesizing MoS_2_ involves sulfurization via thermolysis using MoO_3_ and S powders. However, this approach often yields triangular MoS_2_ domains that overlap, making precise control over layer number and surface coverage challenging (*19*). While recent studies have introduced processes capable of producing MoS_2_ with uniform thickness and minimal domain overlap, many of these methods incorporate buffer layers (*20*), rendering them unsuitable for remote epitaxy.

In this study, a solution-based process was employed, wherein ammonium tetrathiomolybdate ((NH_4_)_2_MoS_4_) precursors were spin-coated onto sapphire and subsequently reduced to MoS_2_ film through thermal processing. To control the thickness uniformity and coverage, the precursor concentration and solvent mixture were optimized (see Methods for further details). Details of MoS_2_ synthesis and optimization process can be found in our previous report (*21*). The thickness uniformity and coverage of the synthesized MoS_2_ were characterized by Raman spectroscopy, in which the number of layers was determined by the spacing (∆_mode_) between the E_2g_ and A_1g_ peaks. Polycrystalline MoS_2_ films synthesized with precursor concentrations of 0.008, 0.012, 0.024, and 0.048 M exhibited ∆_mode_ values of ∼20.91, ∼22, ∼23.12, and ∼ 25.31 cm^−1^, corresponding to mono-, bi-, tri-, and five-layer MoS_2_, respectively (fig. S1A). A top-view image of monolayer MoS_2_ taken by scanning electron microscopy (SEM) in Fig. 2A shows the continuous layer without any triangular domains over a wide area. Furthermore, Raman mapping with diffraction grating with 600 grooves over a 30- by-30 μm^2^ area (Fig. 2B) revealed an average ∆_mode_ of ∼21 cm^−1^, with the maximum and minimum ∆_mode_ measured at 21.3 and 16 cm^−1^, respectively. These values are well within the established range for monolayer MoS_2_ (*20*, *22*–*25*). However, thicker MoS_2_ exhibited more non-uniform thickness (fig. S1B), which could have affected the Ga_2_O_3_ growth. We also confirmed that these MoS_2_ layers do not oxidize during α-Ga_2_O_3_ growths by mist CVD at atmospheric pressure (fig. S1, C and D).

**Fig. 2.**
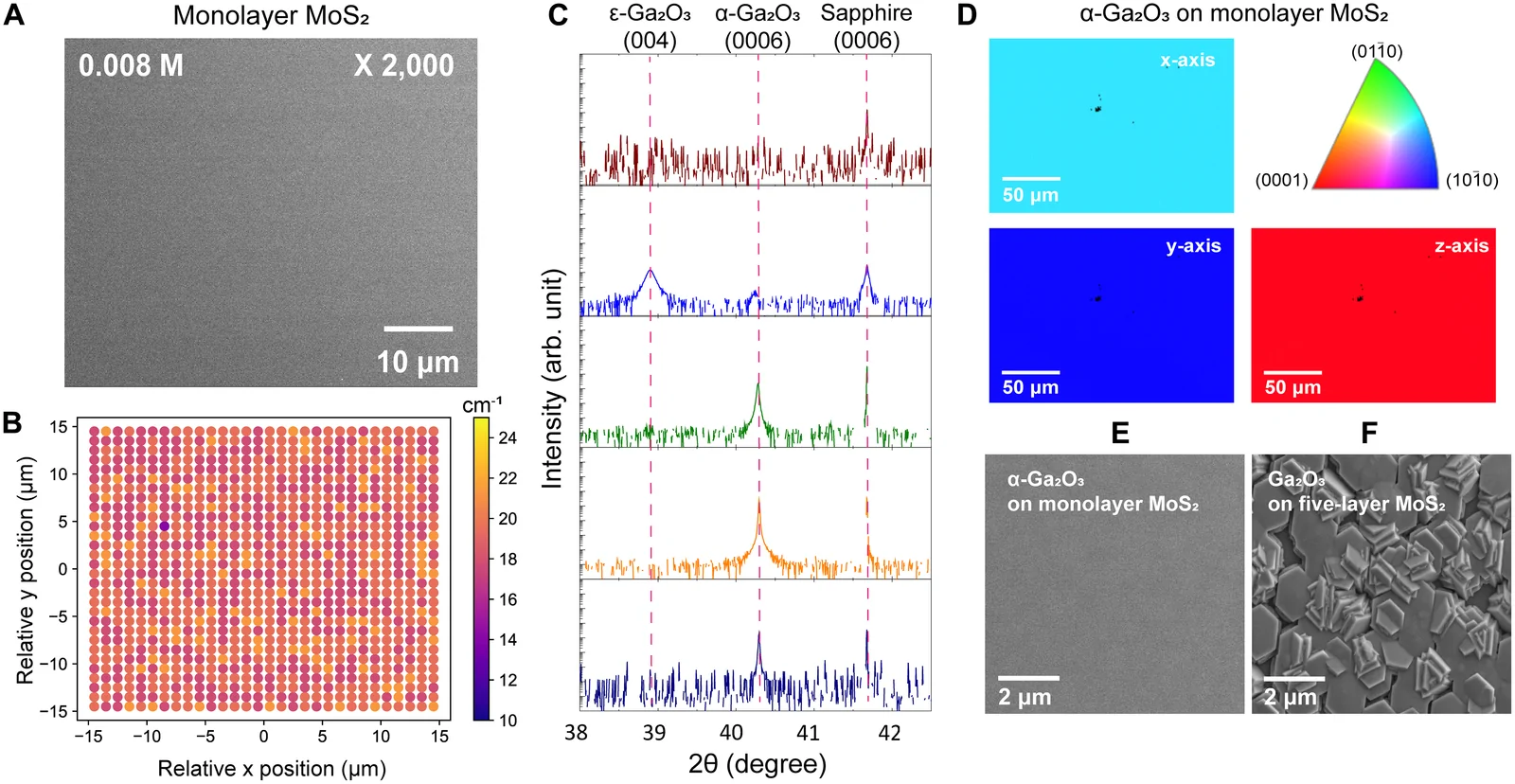
Growth characterizations of MoS_2_ and Ga_2_O_3_. (**A**) Top-view SEM image of monolayer MoS_2_ on sapphire, (**B**) Raman mapping of monolayer MoS_2_ over a 30-by-30 μm^2^ area, with the scale bar indicating the range of ∆_mode_, (**C**) XRD 2θ-ω scans of Ga_2_O_3_ grown on MoS_2_ with varying thickness, (**D**) EBSD analysis of single-crystalline α-Ga_2_O_3_ grown on monolayer MoS_2_, and top-view SEM images of Ga_2_O_3_ films grown on (**E**) monolayer MoS_2_ and (**F**) five-layer MoS_2_.

### α-Ga_2_O_3_ grown on mono- and multi-layer MoS_2_

α-Ga_2_O_3_ was grown at 450°C using mist CVD at a typical growth rate of 0.75 μm/hour. The structural characteristics of the films varied with increasing MoS_2_ layer thickness, as indicated by the x-ray diffraction (XRD) 2θ-ω scans in Fig. 2C. On monolayer MoS_2_, both XRD and electron backscatter diffraction (EBSD) (Fig. 2D) confirmed the formation of highly oriented single-phase α-Ga_2_O_3_, similar to α-Ga_2_O_3_ grown directly on sapphire. Additionally, scanning electron microscopy (SEM) images of the α-Ga_2_O_3_ surface (Fig. 2E) showed no discernible morphological differences compared to growth on bare sapphire. Thus, both XRD and SEM analyses indicate that α-Ga_2_O_3_ on monolayer MoS_2_ exhibits structural characteristics nearly identical to those on bare sapphire. Figure 2C shows that the α-phase is present even on bi- and tri-layer MoS_2_. However, the crystal quality quickly deteriorates (see Fig. 3H), suggesting the growth process is vastly different from the monolayer sample. Details of the growth study are discussed later. Growth on tri-layer MoS_2_ resulted in hexagonal ε-Ga_2_O_3_, coexisting with α-Ga_2_O_3_, as observed in Fig. 2C. On five-layer MoS_2_, the film predominantly exhibited the ε-phase in the form of large hexagonal islands (Fig. 2F).

**Fig. 3.**
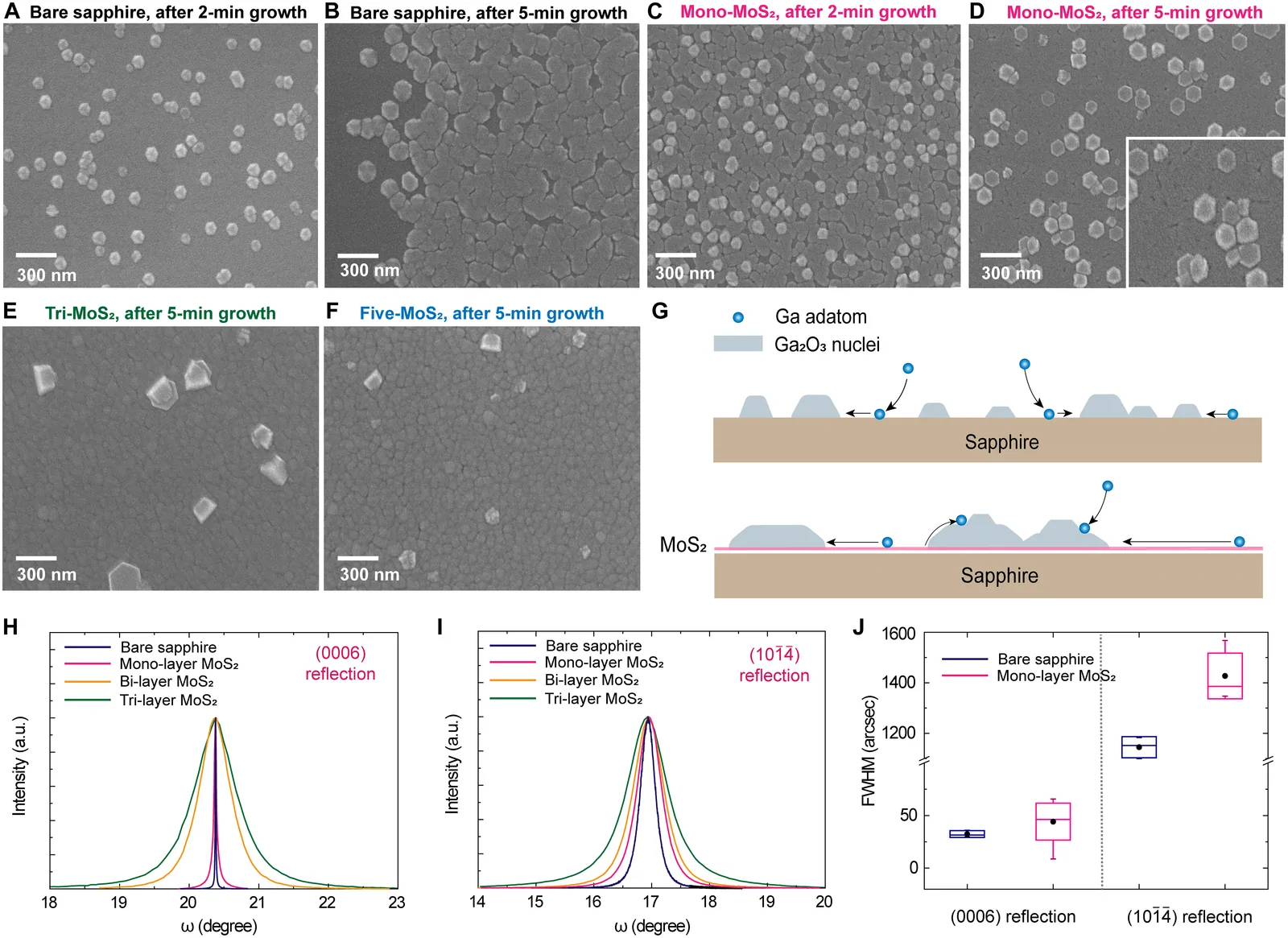
Understanding nucleation and growth of Ga_2_O_3_ on MoS_2_-on-sapphire. Top-view SEM images of α-Ga_2_O_3_ nuclei on bare sapphire after (**A**) 2 minutes and (**B**) 5 minutes of growth. Top-view SEM images of α-Ga_2_O_3_ nuclei on monolayer MoS_2_ after (**C**) 2 minutes and (**D**) 5 minutes of growth with the inset showing the incomplete coalescence underneath top nuclei. (**E**) and (**F**) Top-view SEM images of Ga_2_O_3_ films on tri- and five-layer MoS_2_ after 5 minutes of growth, respectively. (**G**) Schematic illustration of nucleation growth processes of Ga_2_O_3_ on sapphire with and without monolayer MoS_2_, describing fewer but larger rhombohedral nuclei on the MoS_2_ surface. (**H**) and (**I**) XRD rocking curves of the on-axis (0006) and off-axis (10$\bar{1}$4) reflections obtained from the α-Ga_2_O_3_ films. (**J**) Comparison of the average FWHM values of the (0006) and (10$\bar{1}$4) reflections for Ga_2_O_3_ on bare sapphire and monolayer MoS_2_.

According to our density function theory (DFT) calculations, electrons in S atoms are drawn to Al atoms in certain configurations, creating charge-depleted regions on the surface S atoms. This substrate-induced charge redistribution on the MoS_2_ surface is preserved only in the monolayer case. Therefore, when the charge-modulated adsorption landscape is preserved (monolayer case), α-phase nucleation is energetically favored. In contrast, when this interaction is weakened in multilayer MoS_2_, the positional correlation between adsorption sites and the substrate lattice is lost, reducing the energetic preference for α-phase formation and resulting in a shift in phase tendency toward ε-Ga_2_O_3_. The details of the DFT calculations are further discussed below.

At the early stage of growth, we noticed that the nucleation on MoS_2_-on-sapphire was distinctively different from that on bare sapphire and strongly dependent on the number of MoS_2_ layers. After 2 to 5 minutes of growth on bare sapphire, rhombohedral-shape α-Ga_2_O_3_ nuclei were formed and started coalescing on sapphire (Fig. 3, A and B). In comparison, the surface of Ga_2_O_3_ on monolayer MoS_2_ (Fig. 3, C and D) indicates that the nuclei were forming on top of the already coalescing surface, suggesting faster nucleation and growth than the bare sapphire case. Despite the faster nucleation, the well-aligned nuclei suggested the process was still governed by the underlying sapphire. With increasing MoS_2_ thickness, fewer nuclei were observed, and their shapes became increasingly random, likely due to the disordered Ga_2_O_3_ layer underneath (Fig. 3, E and F). In the case of the five-layer MoS_2_, the Ga_2_O_3_ film grew as a film, but its phase could not be determined by XRD, suggesting the film was very disordered (fig. S2, A and C). Prolonged growth on this disordered Ga_2_O_3_ eventually led to the formation of large ε-phase islands (fig. S2, B and C), a phenomenon previously observed in other studies where the ε-phase emerged on an amorphous layer of TiO_2_ (*26*). We attribute the difference in nucleation rate, shown in Fig. 3, A and C, to the change in density of nucleation sites for Ga.

Experimentally, nucleation on monolayer MoS_2_ is characterized by a lower density of well-aligned nuclei, along with secondary nucleation occurring on already coalescing surfaces. In contrast, increasing the MoS_2_ thickness leads to a reduction in nucleation density and more randomly distributed nuclei. Based on our observation and DFT calculations, we propose that the charge-depleted regions on the growth surface create fewer nucleation sites for Ga. This reduction in site density enhances the adatom mobility, thereby promoting the nucleation of new Ga_2_O_3_ microcrystals on top of the coalescing surface, as observed in Fig. 3C. On multilayer MoS_2_, the absence of nucleation sites makes the initial nucleation process more difficult and random.

MoS_2_ could have altered the nucleation kinetics, but our observations suggest that the overall growth rate remained relatively constant with the monolayer MoS_2_. There was no morphological change due to the MoS_2_ layer, but faster nucleation could have attributed to slight increases in the roughness, as confirmed atomic force microscopy (AFM) (fig. S3, A and B). Additionally, AFM imaging of Ga_2_O_3_ grown on tri-layer MoS_2_ (fig. S3C) shows a much rougher surface with significant morphological changes, consistent with the observed change in the nucleation process.

The α-Ga_2_O_3_ films with thicknesses of 0.5 ∼ 1.5 μm grown on sapphire by our mist CVD typically exhibits full-width-at-half-maximum (FWHM) values of 40 ∼ 70 and 900 ∼ 1300 arcsec for the (0006) and (10$\bar{1}\bar{4}$) reflections in rocking curve mode, respectively. These values are comparable to those reported in the literature (*27*). The relatively low FWHM values for the on-axis (0006) reflection suggest that the domains are highly oriented along the c-axis, with minimal tilt. In contrast, the higher FWHM values for the (10$\bar{14}$) reflection can be interpreted as a significant density of edge- and mixed-type dislocations typically with a Burgers vector $b_{e}=\frac{1}{3}\langle 2\bar{1}\bar{1}0\rangle$ (*28*), where the dislocations are mainly produced by the mismatches in lattice constants and coefficients of thermal expansion (CTE) between α-Ga_2_O_3_ (CTE ∼ 9.35 × 10^−6^°C^−1^) and sapphire (CTE ∼ 8.11 × 10^−6^°C^−1^) (*29*).

Due to the large lattice mismatch of approximately 4.6% between α-Ga_2_O_3_ and sapphire, the critical thickness of α-Ga_2_O_3_ on sapphire is expected to be less than 10 nm (*29*, *30*). But unlike conventional epitaxy, remote epitaxial films are possibly less sensitive to the lattice mismatch, as they can relax elastically due to their weak vdW interaction with the underlying 2D layer (*31*). The strain mapping analysis via cross-sectional high-angle annular dark field (HAADF) scanning transmission microscopy (STEM) suggests that α-Ga_2_O_3_ is essentially strain-free near the interface (fig. S4). While more macroscopic reciprocal space mapping (fig. S5) indicates that the films are fully relaxed on a film scale, a traditional covalent interface would typically retain significant residual strain near the interface due to the lattice and CTE mismatches. Specifically, the CTE mismatch would be expected to induce localized tensile strain during the cooling process. The observation that the Ga_2_O_3_ remains strain-free while coupled to the substrate suggests interfacial “slippage” facilitated by van der Waals (vdW) interactions. This localized relaxation—occurring despite the substrate’s thermal contraction—provides compelling evidence for a remote epitaxial mechanism and demonstrates the mechanical decoupling afforded by the MoS_2_ interlayer.

On average, however, the α-Ga_2_O_3_ films on monolayer MoS_2_/sapphire exhibited a slight increase in FWHM values of both (0006) and (10$\bar{1}\bar{4}$) reflections compared to those grown directly on sapphire (see Fig. 2, H through J). In the remote epitaxy of GaN on graphene/SiC, S. Lee *et al*. reported a reduction in crystal quality, which was primarily attributed to the requirement of a thick, low-temperature GaN buffer to achieve full film coalescence (*32*). This suggests that the low surface energy of vdW surface modifies the growth kinetics. Given that there is no buffer layer in our case, it is possible that the nucleation process becomes more dependent on the growth conditions, which influence the crystal quality.

Although beam-induced damage of MoS_2_ precluded high-magnification imaging of the defect core structures, the current STEM image in fig. S6 clearly indicates that the dislocations are generated within the Ga_2_O_3_ layer rather than being extended from the substrate. The threading dislocation density is estimated to be on the order of 10^10^–10^11^ cm^−2^, which is in good agreement with the dislocation density estimated by the off-axis FWHM values obtained from XRD. This observation is important, as it suggests that the defect density is affected by the initial nucleation and coalescence kinetics on the MoS_2_ surface. Additionally, it remains possible that localized imperfections, such as grain boundaries inherent in polycrystalline MoS_2_, act as preferential sites for defect nucleation. Although a direct spatial correlation between the MoS_2_ grain boundaries and Ga_2_O_3_ defects could not be established within the examined regions, previous study on remote epitaxy has indicated that 2D template defects and grain boundaries can significantly alter the chemical reactivity and surface potential, thereby degrading the overlayer crystallinity (*33*). Therefore, the grain boundaries in polycrystalline MoS_2_ may have contributed to the slightly lower crystal quality compared to growth on a bare sapphire substrate.

This kinetics-dependent growth is further supported by growth experiments in which the growth temperature was varied from 450 to 470°C. Given the phase control is temperature-sensitive (*34*), the explored growth window may have been limited to achieve optimal results. Nevertheless, within this range, increasing the temperature led to overall decrease in FWHM values. Specifically, the discrepancy in off-axis FWHM values between the bare sapphire and MoS_2_ samples was reduced (fig. S7). Overall, the crystal quality of remote epitaxial films is possibly more sensitive to growth conditions due to the weakly interacting vdW surface.

When bilayer MoS_2_-on-sapphire was used as the substrate, the FWHM values increased significantly, exceeding 1000 and 2000 arcsec for the (0006) and (10$\bar{1}\bar{4}$) reflections, respectively, suggesting a substantial degradation in crystal quality (Fig. 3, H and I and table S1) due to the transition from remote to vdW epitaxy. Cross-sectional transmission electron microscopy (TEM) images of Ga_2_O_3_ grown on bi- and tri-layer MoS_2_ are presented in fig. S9, A and B. In both cases, fast Fourier transform patterns from the imaged areas reveal amorphous Ga_2_O_3_ intermixed with the α-phase. As the MoS_2_ thickness increased further, the crystal quality of Ga_2_O_3_ continued to deteriorate progressively. For the ε-phase grown on five-layer MoS_2_, the FWHM broadening became so excessive that reliable measurements could not be obtained.

To examine the interface regions in greater detail, cross-sectional scanning TEM (STEM) was performed on single-crystalline α-Ga_2_O_3_ grown on monolayer MoS_2_/sapphire. The low-magnification STEM image (Fig. 4A) shows a laterally continuous MoS_2_ layer over an extended region. Combined with the Raman mapping results (Fig. 1B), this observation strongly suggests the solution-processed monolayer MoS_2_ forms uniformly over a wide area without detectable pinholes or structural discontinuities. The crystallographic alignment was further confirmed by selected area electron diffraction (SAED) acquired along the [$\bar{1}2\bar{1}0$] zone axis (Fig. 4B), showing two well-aligned sets of diffraction spots from α-Ga_2_O_3_ and sapphire, thereby confirming the epitaxial relationship between the film and the substrate.

**Fig. 4.**
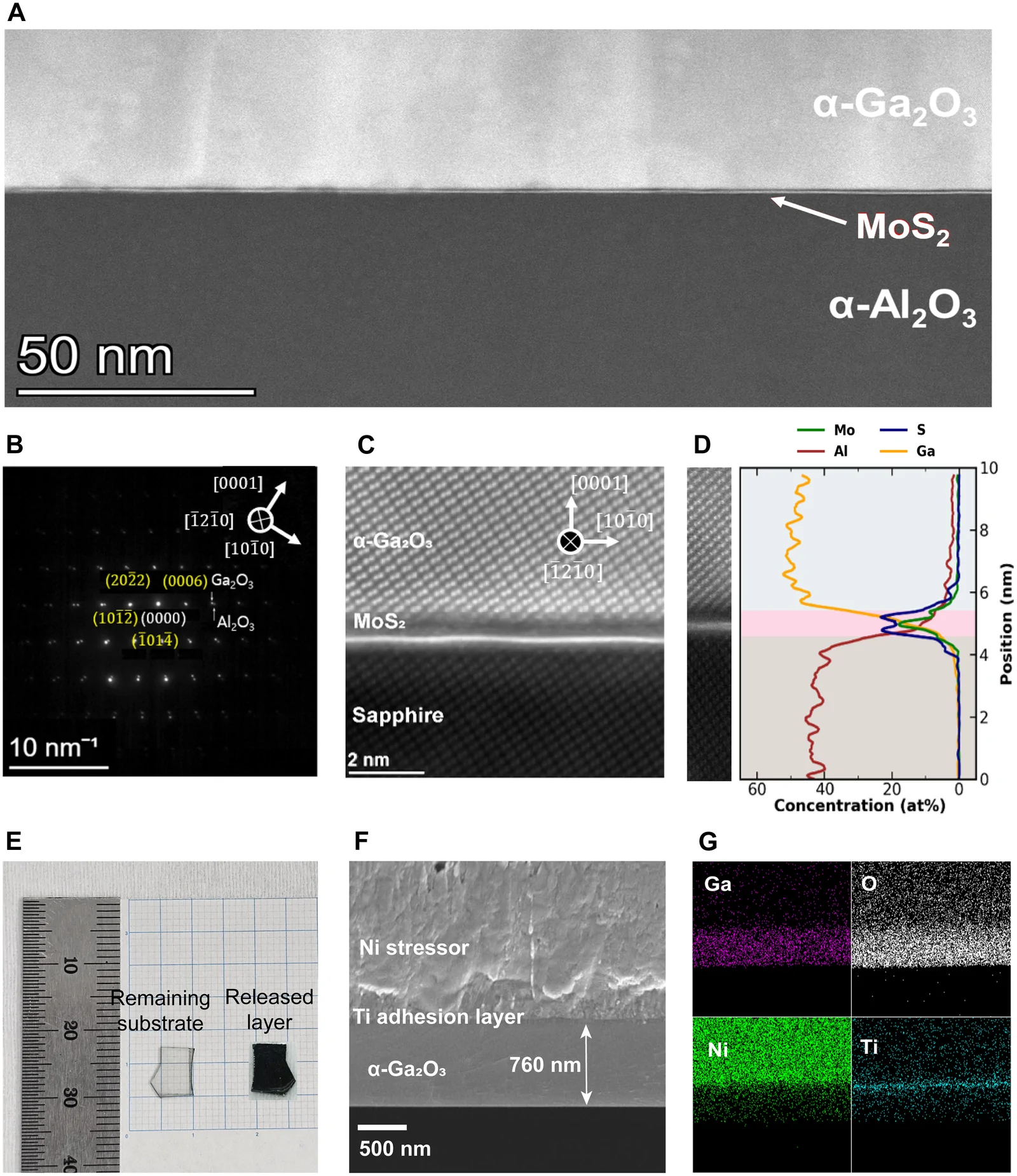
Microstructural analysis and exfoliation of remote-epitaxially grown α-Ga_2_O_3_. (**A**) Low-magnification cross-sectional HAADF-STEM image of the MoS_2_ layer over an extended lateral region (>200 nm). (**B**) SAED pattern acquired along the [$\bar{1}2\bar{1}0$] zone axis. (**C**) High-magnification cross-sectional STEM image showing the MoS_2_ interlayer and α-Ga_2_O_3_. (**D**) EDS line profile across the interface region in (C). (**E**) Photograph of exfoliated Ni-coated Ga_2_O_3_ on thermal release tape alongside the remaining substrate. (**F**) Cross-sectional SEM image showing the Ni stressor, Ti adhesion layer, and Ga_2_O_3_. (**G**) Corresponding EDS elemental mapping of Ga, O, Ni, and Ti.

More importantly, the high-magnification STEM image of the interface (Fig. 4C) reveals that the single-crystalline α-Ga_2_O_3_ appears to have grown in direct registry with the sapphire, despite the presence of the intermediate MoS_2_ layer. Notably, the polycrystalline nature of MoS_2_ did not compromise the single-crystallinity of the Ga_2_O_3_ epilayer. Strain mapping of the α-Ga_2_O_3_ above MoS_2_ also shows no localized high-strain regions associated with structural defects within the analyzed region (fig. S4). Additionally, atomic-resolution HAADF and annular bright-field (ABF)-STEM images (fig. S8, A and B), along with their corresponding integrated intensity profiles (fig. S8C), suggest that the initial layer on the MoS_2_ interlayer is the Ga atom layer, which corroborates the DFT predictions. The monolayer nature of MoS_2_ was further confirmed by the two sharp peaks of Sulfur and a single Molybdenum peak in energy-dispersive spectroscopy (EDS) analysis, also shown by a sharp intensity in the line profile extracted across the interface (Fig. 4D). Collectively, this comprehensive analysis of the interface region provides structural evidence for remote epitaxy of α-Ga_2_O_3_ on MoS_2_.

As the layer count increased from monolayer to multilayer configurations (bi-, tri-, and five-layers), the Ga_2_O_3_ structure became progressively more disordered (fig. S9). Both the XRD and STEM analyses consistently demonstrated that monolayer MoS_2_ facilitates remote epitaxy—a conclusion further validated by successful mechanical exfoliation and DFT calculations discussed in subsequent sections.

### Exfoliation of α-Ga_2_O_3_

One of the key features of remote epitaxy is that the epitaxial film can be easily exfoliated with minimal mechanical force, which can be typically induced by depositing a thick layer of Ni (*1*, *35*). By applying a Ni stressor, we successfully exfoliated a centimeter-sized α-Ga_2_O_3_ film from a monolayer MoS_2_-on-sapphire substrate without any damages (Fig. 4, E to G). The exfoliated α-Ga_2_O_3_ membrane was crack-free and devoid of any structural defects or features, as expected from remote-epitaxially grown films. The substrate side was similarly clean, with negligible traces of Ga, indicating that no Ga_2_O_3_ remained on the surface (fig. S10). These results indicate that the exfoliation occurred at the weakest point – the interface, which was held together by relatively weak vdW interactions.

### Understanding growth mechanism

Experimental results demonstrated that high-quality single-crystal α-Ga_2_O_3_ films were successfully synthesized via remote epitaxy using a polycrystalline monolayer of MoS_2_ as a 2D interlayer. Considering previous remote-epitaxy studies that employed single-crystalline and monatomic graphene (*1*, *31*, *33*), the polar-bonding nature of the intervening MoS_2_ layer suggests that a distinctly different remote-epitaxy mechanism may be required to explain the present system. It is well known that graphene-based remote epitaxy relies on the “lattice transparency” of a one-atom-thick carbon layer, which allows the atomic potential of substrate to influence the growing film despite the presence of graphene (*3*, *36*). Due to the weak vdW potential of monolayer graphene, it cannot fully screen the stronger electrostatic field of a polar substrate, enabling adatoms to register to the substrate lattice. In contrast to the purely in-plane sp^2^ bonds with no permanent dipole for graphene, Mo atoms in MoS_2_ have out-of-plane 4d orbitals that overlap with 3p orbitals of sulfur, forming polar covalent bonds oriented perpendicular to the MoS_2_ monolayer (*37*–*41*). Furthermore, MoS_2_ is not as “electrically transparent” as graphene due to its greater thickness.

To elucidate the underlying mechanism enabling freestanding single-crystal α-Ga_2_O_3_ growth via MoS_2_ interlayer, we conducted DFT calculations. Figure 5A presents one of the lowest-energy heterostructure models identified in this study, consisting of an atomically optimized Al_2_O_3_ substrate, a monolayer MoS_2_, and an epitaxial α-Ga_2_O_3_ overlayer. As a first step, we examined the optimal MoS_2_/Al_2_O_3_ interface configurations in the absence of the α-Ga_2_O_3_ layer to identify the most stable interfacial arrangements. Multiple low-energy configurations with varying relative in-plane orientations were found, as illustrated in Fig. 5B and fig. S11, all differing in formation energy by only ∼3 meV. Despite these small energy differences, the electronic charge distribution within the MoS_2_ layer varied significantly depending on the specific interfacial alignment. Notably, configurations I and II (see Fig. 5B) exhibited pronounced charge depletion on the top surface of the MoS_2_ layer, influenced by the underlying Al_2_O_3_ substrate. This depletion does not occur uniformly across all top sulfur atoms (referred to as S_top_ in Fig. 5C), but rather selectively, depending on the geometric alignment among the S_top_ atoms, the lower sulfur atoms (S_bott_), and the topmost aluminium atoms (Al_top_) of the Al_2_O_3_ substrate. More precisely, significant charge depletion occurs when the Al_top_, S_bott_, and S_top_ atoms are nearly colinear, as shown in Fig. 5C. When the charge depletion regions are formed, the charge accumulation regions also appear (see Fig. 5B). Deviations from this ideal colinear atomic alignment result in reduced charge depletion - or even local charge accumulation when the deviation is substantial.

**Fig. 5.**
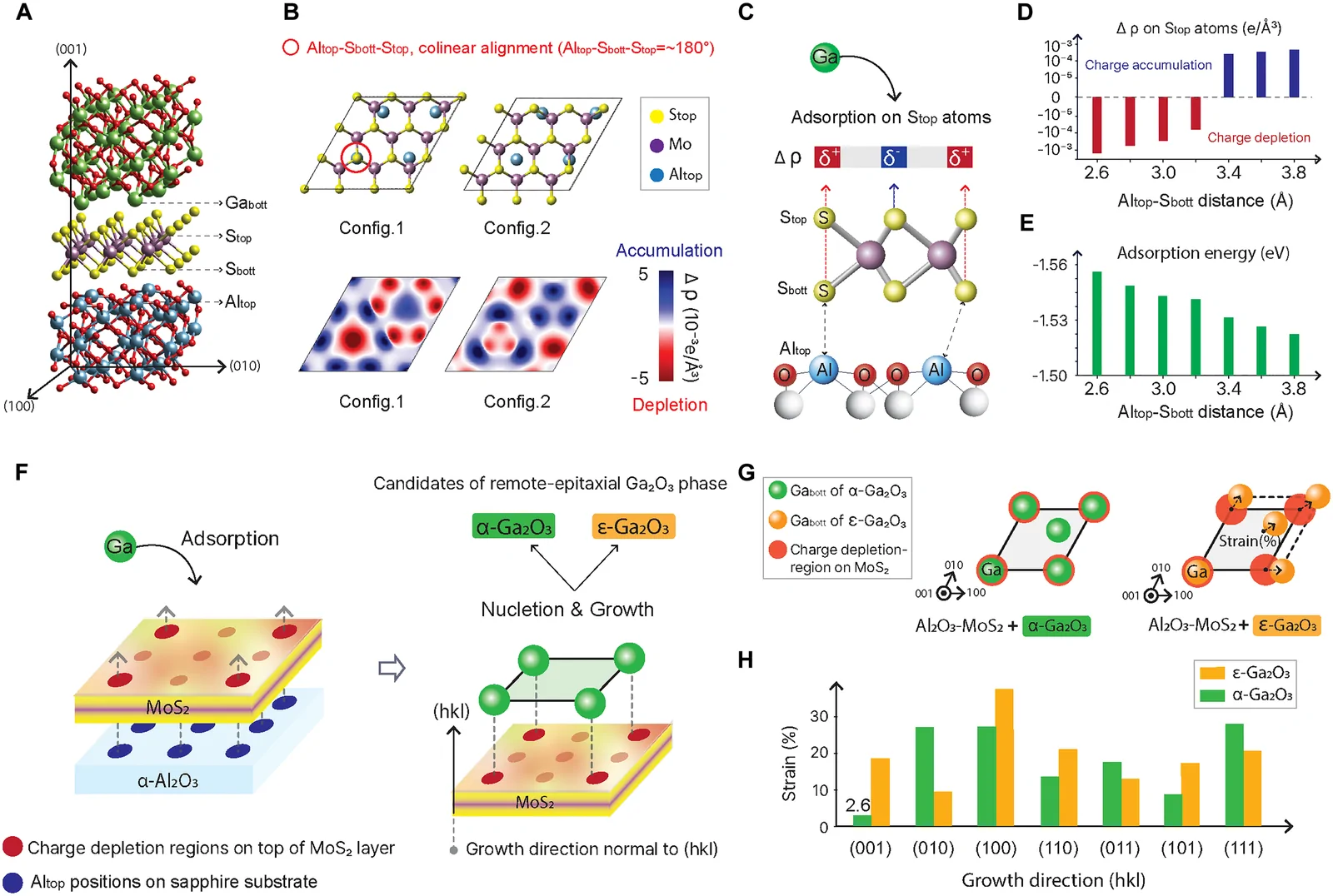
DFT study on the mechanism of remote epitaxy of Ga_2_O_3_ via a MoS_2_ 2D layer. (**A**) A representative heterostructure model consisting of α-Ga_2_O_3_/MoS_2_/α-Al_2_O_3_ layers. (**B**) (top) C-axis view of Al_2_O_3_–MoS_2_ interface configurations 1 and 2 (bottom). Charge density difference maps (Δρ), showing electron depletion (δ+, red) and accumulation (δ−, blue). (**C**) Schematic illustrating the correlation between Al_top_ positions/distances and the charge density difference (Δρ). As colinearity increases (or equivalently, as the Al_top_-S_bott_ distance decreases), larger charge depletion is observed on the S_top_ atoms in MoS_2_. (**D**) Calculated charge density difference on the S_top_ atom as a function of the Al_top_-S_bott_ distance. (**E**) Adsorption energy (in eV) of a Ga adatom as a function of the Al_top_-S_bott_ distance, showing that larger charge depletion corresponds to stronger adsorption. A similar dependence of adsorption strength on the Al_top_-S_bott_ distance is observed for the Ga(H_2_O)_3_ complex (see fig. S12). (**F**) (left) Schematic of how the positional information of Al_top_ atoms is transmitted to S_top_ atoms, forming a 2D charge-depletion pattern. (right) α- and ε-Ga_2_O_3_ polymorphs as possible epitaxial phases. (**G**) Schematic showing the positional coherence of Ga_bott_ atoms with charge-depleted S_top_ sites. (**H**) Computed strain values resulting from lattice matching between the MoS_2_ layer and various crystallographic orientations of the α and ε phases (hkl = 001, 010, 100, 110, 101, 011, and 111). The α-Ga_2_O_3_ phase grown along the (001) orientation exhibits the lowest strain (2.3%).

Given that the degree of collinearity is closely related to the Al_top-_S_bott_ distance (fig. S12), a shorter Al_top-_S_bott_ distance also correlates strongly with increased charge depletion on S_top_, indicating that the strong influence of Al_top_ is mediated via S_bott_ to the MoS_2_ surface. This substrate-induced charge redistribution is not unique to a single configuration; similar trends are also observed across other Al_2_O_3_/MoS_2_ heterostructure models (fig. S13). Consequently, the majority of Al_top_ atoms on the Al_2_O_3_ substrate effectively transmit their positional information through the MoS_2_ layer, generating a patterned array of charge-depleted S_top_ atoms on its surface, as schematically visualized in Fig. 5F. Remarkably, this pattern mirrors the crystallographic symmetry of the underlying Al_2_O_3_ substrate. Such charge polarization on S_top_ atoms is observed only in monolayer MoS_2_ and is absent in the bilayer MoS_2_ structures examined (fig. S14), indicating that the substrate ‘transparency’ necessary for remote epitaxy may operate only in monolayer MoS_2_.

The next question is how this Al_2_O_3_-induced charge redistribution in the S_top_ atoms correlates with the single-crystal growth of the Ga_2_O_3_ epitaxial layer. To investigate this, we calculated the adsorption energies of Ga adatoms on the MoS_2_ surface. We found that stronger adsorption occurs at sites exhibiting greater charge depletion (see comparison in Fig. 5, D and E). A similar adsorption trend is observed for the hydrated Ga(H_2_O)_3_ complex (fig. S15), which has been proposed as one of the initial adsorbing Ga species in the mist CVD process (*42*). From a kinetic perspective, higher adsorption energy implies longer residence times for Ga adatoms at these S_top_ sites, which can facilitate nucleation. Therefore, it is reasonable to assume that, during the growth of the epitaxial Ga_2_O_3_ layer, nucleation preferentially occurs at the charge-depleted S_top_ sites due to their stronger interaction with Ga adatoms. If this assumption holds, then the positions of Ga_bott_ atoms should align closely with these charge-depleted regions to enable stable single-crystal growth. To test this, we compared the atomic positions of Ga_bott_ in two Ga_2_O_3_ polymorphs (α and ε phases) with the locations of charge-depleted S_top_ atoms, as schematically illustrated in Fig. 5, F and G. The corresponding strain values for various growth orientations of each phase are also shown in Fig. 5H. This analysis reveals that α-Ga_2_O_3_ grown along the (001) direction exhibits the best alignment with the charge-depleted sites and the lowest interfacial strain, making it the more favorable epitaxial phase on the MoS_2_ layer. This finding is consistent with our experimental observations. Ultimately, it is this localized charge redistribution on the MoS_2_ surface, induced by Al_2_O_3_-MoS_2_ interactions, that governs the crystallization kinetics of the Ga_2_O_3_ epitaxial layer. Despite being significantly thicker than graphene, MoS_2_ functions as a “transparent” 2D layer in a similar manner, transmitting the structural information of the substrate to the growing film and thereby enabling the remote epitaxial growth of single-crystal α-Ga_2_O_3_.

Finally, the small differences in formation energy among the various Al_2_O_3_-MoS_2_ interface configurations (tables S2 and S3) suggest that the difference in energy barriers for MoS_2_ nucleation on the Al_2_O_3_ substrate are similarly small across these configurations. Consequently, MoS_2_ grains with orientations other than configuration I, the lowest-energy configuration, can form with non-negligible probabilities, resulting in the experimentally observed polycrystalline MoS_2_ layer. However, despite the presence of varied in-plane orientations within this polycrystalline MoS_2_ film, the charge redistribution at the MoS_2_-substrate interface remains highly selective and consistently tied to the positions of the Al_top_ atoms on the Al_2_O_3_ substrate, as shown in Fig. 5B and fig. S13. This indicates that the underlying substrate can still impose a spatially patterned electrostatic influence across different MoS_2_ grains. Therefore, a plausible explanation for the observed single-crystal α-Ga_2_O_3_ epitaxial growth on polycrystalline MoS_2_ is the preferential nucleation and subsequent growth of Ga_2_O_3_ at these substrate-imprinted, charge-depleted sites, regardless of the grain orientation of the underlying MoS_2_.

As outlined in note S1, this charge-mediated remote epitaxy is not expected to occur on an O-terminated sapphire surface, as such termination would promote direct covalent bonding between O and S. Although no thermal treatment was applied to the sapphire substrate prior to spin-coating the precursor solution, the spectra taken by x-ray photoemission spectroscopy (XPS) from monolayer MoS_2_ on sapphire (see fig. S16, A to C) predominantly indicate stoichiometric MoS_2_, with no significant chemical interaction between MoS_2_ and the sapphire surface.

The only deviation is found in the S 2p signal in fig. S16B, which exhibits a peak at 168 ∼ 169 eV corresponding to S-O bonding. The oxygen in these S–O species likely originates from the sapphire surface or from adsorbed hydroxyl groups, which can be corroborated by Al-OH bonding shown in fig. S16C. The localized formation of S-O bonds may have influenced the remote epitaxy process, the subsequent exfoliation behavior, as well as the crystal quality of epitaxial α-Ga_2_O_3_.

To elucidate the role of S-O bonding, remote epitaxy was attempted on monolayer MoS_2_ synthesized on O_2_-plasma-treated sapphire (see note S2 and fig. S17 for more details). The plasma treatment was utilized to deliberately induce surface hydroxylation. Notably, α-Ga_2_O_3_ growth was entirely suppressed on the monolayer template following the surface treatment. These results, corroborated by DFT calculations, substantiate the viability of charge-mediated remote epitaxy of single-crystalline α-Ga_2_O_3_ on MoS_2_.

We successfully demonstrated remote epitaxy of centimeter-scale α-Ga_2_O_3_ using polycrystalline MoS_2_ as a 2D vdW layer. Given that metastable α-Ga_2_O_3_ can only be grown on substrates with matching crystal structure, the monolayer MoS_2_ was sufficiently thin to allow the substrate to dictate the nucleation process. However, as the MoS_2_ thickness increased, the nucleation process was no longer controlled by the substrate, resulting in a more disordered Ga_2_O_3_ film. Our DFT calculations revealed that the lowest energy configuration for Ga adsorption is achieved when Al directly aligns with the bottom S and Ga aligns with the top S. This results in an Al(δ^+^)-S(δ^−^)-S(δ^+^) arrangement that forms the basis for Ga_2_O_3_ nucleation. The concept of charge-mediated remote epitaxy can be extended to a wide range of materials, enabling rational material selection based on inter- and intra-atomic interactions to achieve optimal growth and exfoliation.

Once α-Ga_2_O_3_ is fabricated as a free-standing membrane, it can be integrated into a variety of platforms at the materials level. As previously mentioned, remote epitaxial α-Ga_2_O_3_ can significantly simplify thermal management. Furthermore, this free-standing Ga_2_O_3_ enables the development of an additional key device class - power electronics - that can be seamlessly integrated with silicon and other compound semiconductors, such as gallium arsenide and gallium nitride. This advancement paves the way for multifunctional electronic systems capable of performing logic, communication, and power management on a single chip (*43*–*45*). Traditionally, integration of diverse devices is only possible at the chip level, which increases the overall footprint and introduces latency issues. The high degree of flexibility afforded by remote epitaxy offers exciting opportunities for advancing next-generation wide-bandgap semiconductor technologies.

## MATERIALS AND METHODS

### 2D polycrystalline MoS_2_ synthesis

Polycrystalline MoS_2_ was synthesized by sulfurization of a precursor solution coated on c-plane sapphire substrates. Prior to spin coating, a substrate was sequentially cleaned by sonication in acetone, isopropanol (IPA), and deionized water. The precursor solution contained (NH_4_)_2_MoS_4_, dissolved in a mixed solvent system of dimethylformamide, n-butylamine and ethanolamine (*21*). This solvent combination was employed to ensure the uniform coating of the precursor solution by controlling the viscosity and preventing the formation of MoS_4_^2−^ clusters during dissolution (*46*).

After spin coatings, samples were soft-baked at 150°C for 30 minutes to remove solvents. The synthesis involved two annealing steps: reduction of (NH_4_)_2_MoS_4_ to MoS_3_ under N_2_/H_2_ flow at <1 Torr and 300°C for 10 minutes, and conversion to MoS_2_ using molten sulfur under N_2_ atmosphere at 600–700 Torr and 900°C for 1 hour.

### α-Ga_2_O_3_ thin film growth

The α-Ga_2_O_3_ thin film was grown using the mist CVD method. Mist CVD is a deposition technique in which the precursor solution is atomized into a mist and transported to the reactor, typically under atmospheric pressure, to facilitate the growth of single-crystal Ga_2_O_3_ (*42*). In this study, a 0.02 M solution of gallium acetylacetonate (Ga(acac)_3_) was prepared in deionized water with added HCl to enhance solubility. The solution was then atomized using a 1.7 MHz ultrasonic transducer and delivered into the reactor by N_2_ carrier gas. To prevent oxidation of MoS_2_ during the temperature ramp-up, an N_2_ ambient was established in the reactor prior to growth. Typical growth temperature and rate for α-Ga_2_O_3_ were 450°C and approximately 700 nm/hour, respectively. To investigate the influence growth conditions on remote epitaxy on MoS_2_, the growth temperature was varied from 420 to 470°C, and the film thickness was controlled in the range of 100 to 700 nm.

### α-Ga_2_O_3_ thin film exfoliation

The exfoliation of the α-Ga_2_O_3_ thin film was carried out using a Ti/Ni stressor. First, the sample with the grown Ga_2_O_3_ thin film was cleaned sequentially with acetone, IPA, and deionized water. A 50 nm Ti adhesion layer was then deposited using an e-beam evaporator to aid in the deposition of the stressor layer. Subsequently, a 3.54 μm Ni stressor layer, which induces tensile stress to facilitate separation, was deposited using DC sputtering. Afterward, a thermal release tape was applied to exfoliate the α-Ga_2_O_3_ thin film. Additionally, SEM EDS analysis was performed on both the exfoliated thin film and the remaining substrate.

### Material characterizations

Material characterization employed multiple techniques: Raman spectroscopy for MoS_2_ thickness determination (Renishaw inVia Reflex) and mapping (HORIBA LabRAM HR Evolution), x-ray diffraction for structural analysis (Panalytical X’pert^3^ MRD systems), and comprehensive surface analysis including SEM (JEOL JSM-6701F), EBSD (Hitachi SU8230), and AFM (Park Systems NX20). Cross-sectional microstructure was examined using TEM/STEM following FIB specimen preparation, with high-resolution imaging at 200 kV (FEI Titan G2) and analytical microscopy at 300 kV (Thermo Fisher Themis Z). XPS analysis of the MoS_2_ on sapphire surface was performed using a NEXSA system (Thermo Fisher Scientific). The excitation source was mono chromatic Al Kα (energy: 1486.6 eV) with a spot size of 400 μm. For fitting of both Mo and O peaks were fitted with symmetric Voigt functions.

### Computational details

The first-principles calculations were performed using the projected augmented wave (PAW) plane-wave basis, implemented in the Vienna ab initio simulation package (VASP) (*47*). An energy cutoff of 520 eV was employed and the atomic positions were optimized until the maximum force on each of them was less than 0.01 eV/Å. The hetero-structures of Al_2_O_3_ and MoS_2_ were modeled using k-point grids of 8x8x1. The Perdew-Burke-Ernzerhof (PBE) functional (*48*) was employed for geometrical optimization and electronic structure calculations. For the van der Waals corrections, Grimme’s DFT-D3 method (*49*) based on a semiempirical GGA-type theory was utilized. The slab models had dangling bonds on the vacuum surface, which were terminated with pseudo-hydrogen atoms with appropriate fractional charges to prevent the generation of surface states. To determine the vacuum level, dipole corrections were introduced to compensate for the artificial dipole moment at the open ends (20 Å vacuum space along the c-axis) arising from the periodical boundary condition imposed in these calculations.

## Supplementary Materials

### This PDF file includes:

Figs. S1 to S17 Tables S1 to S4 Notes S1 and S2 References
