# Supplementary material for: Charge-mediated remote epitaxy of Ga2O3 via MoS2
Source: Sci Adv. 2026 Oct 16;12(42):eaee9510. doi: 10.1126/sciadv.aee9510 (PMC13674503; doi:10.1126/sciadv.aee9510)
Supplement: Supplementary file 1 [file sciadv.aee9510_sm.pdf]

Science Advances
AAAS

Supplementary Materials for

Charge-Mediated Remote Epitaxy of Ga_2O_3 via MoS_2

Gyeong Ryul Lee *et al.*

Corresponding author: Celesta S. Chang, celesta@snu.ac.kr; Hyun S. Kum, hkum@yonsei.ac.kr; Taehoon Lee, thl@knu.ac.kr; Roy B. Chung, roy.b.chung@knu.ac.kr

Sci. Adv. **12**, eaee9510 (2026)
DOI: 10.1126/sciadv.aee9510

This PDF file includes:

Figs. S1 to S17
Tables S1 to S4
Notes S1 and S2
References

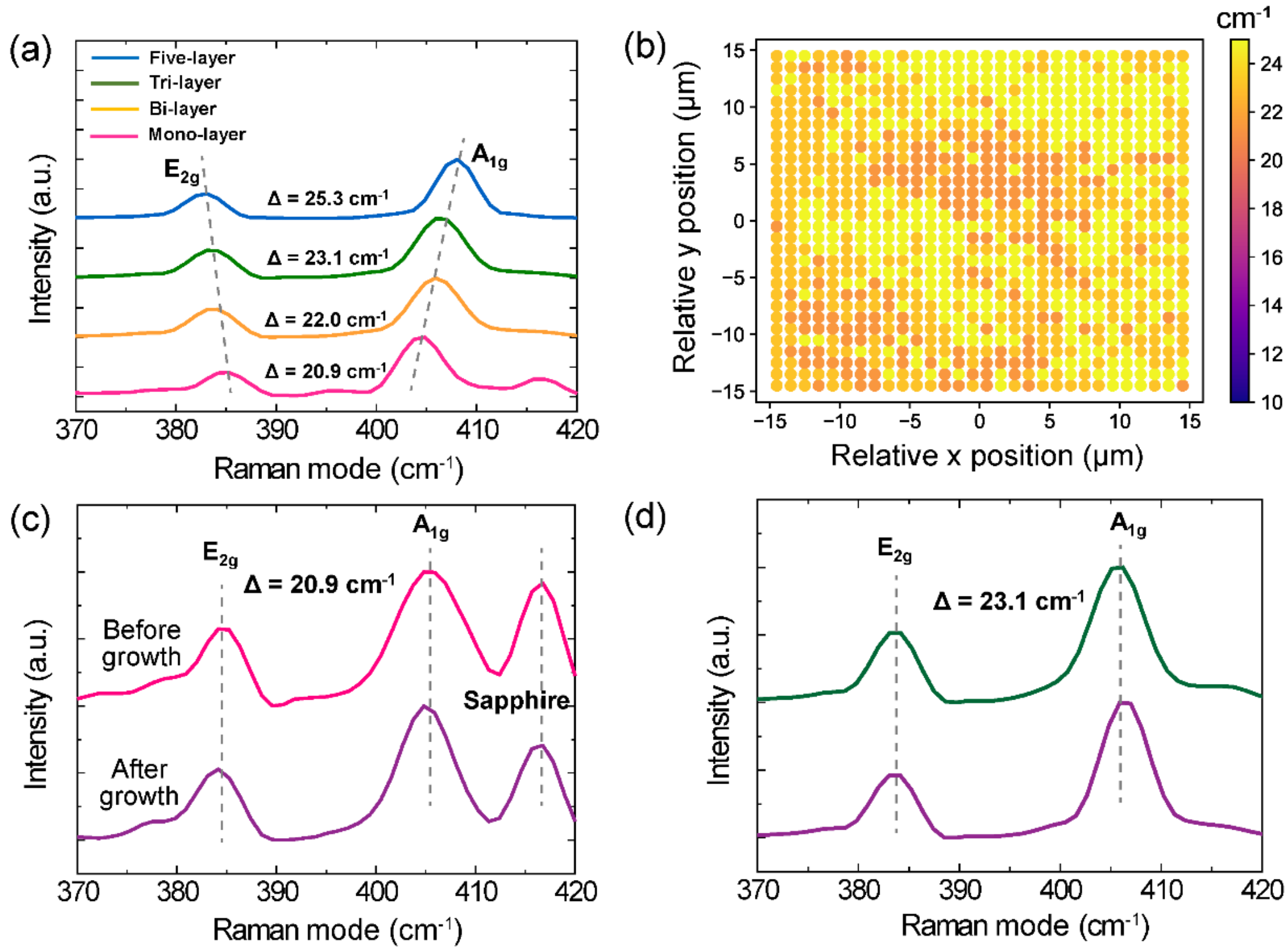

Supplementary Figure 1. Raman spectroscopic and mapping analyses of MoS_2. (a) Raman spectra of mono-, bi-, tri-, and five-layer MoS_2, with layer thickness estimated by the peak shift between E_{2g} and A_{1g} vibrational modes. (b) Raman mapping of 5-layer MoS_2 over a 30-by-30 μm^2 area. Raman spectra of (c) monolayer and (d) five-layer MoS_2 before and after a 5-minute growth of Ga_2O_3 in mist CVD at 450 °C.

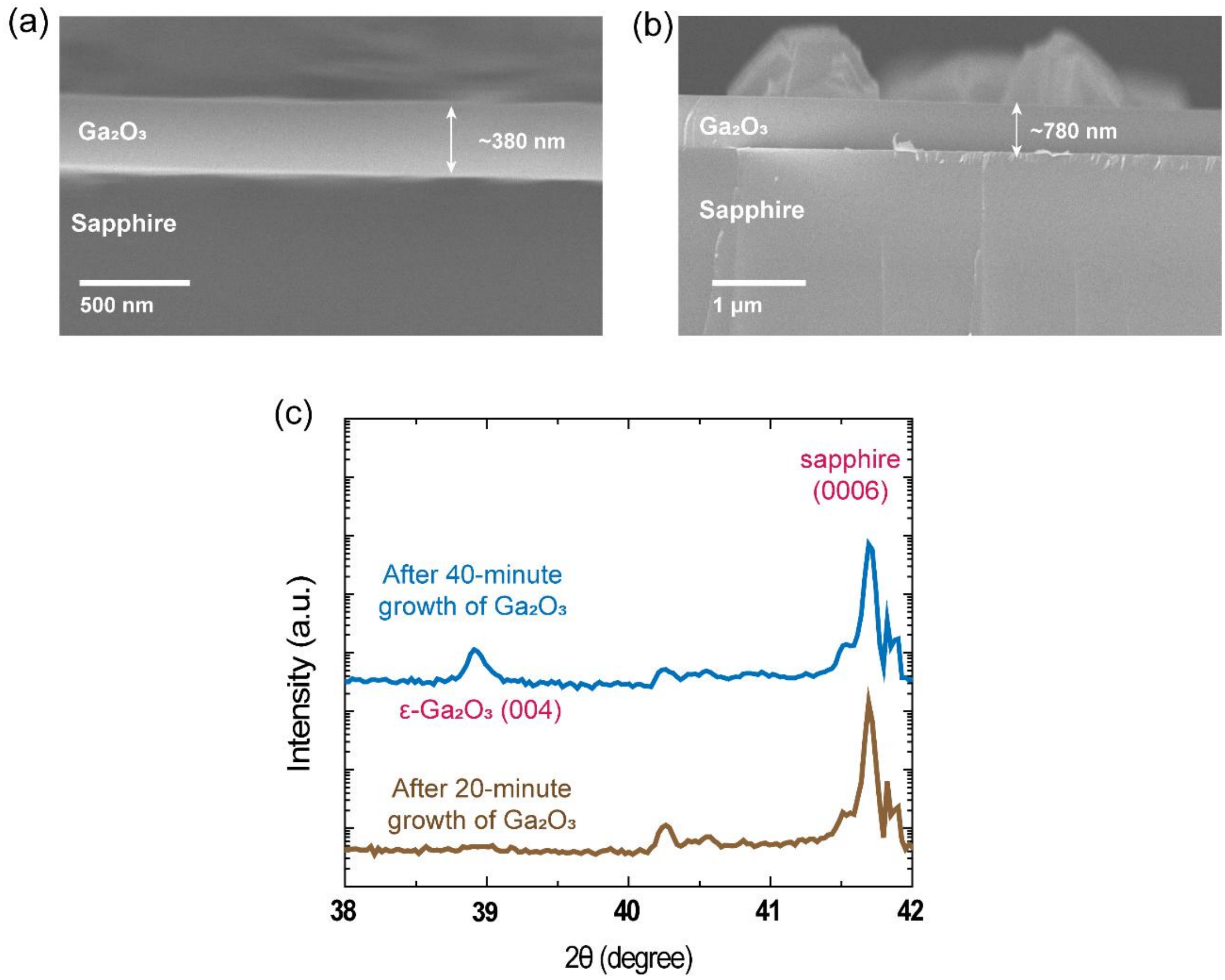

Supplementary Figure 2. Cross-sectional morphology and crystalline structures of Ga_2O_3 on 5-layer MoS_2. (a) Cross- sectional SEM image after 20 minutes of growth, showing a relatively thick layer of Ga_2O_3, whose structure could not be identified by XRD. (b) Cross-sectional SEM image after 40 minutes of growth, revealing the emergence of large ε-Ga_2O_3 islands atop the disordered Ga_2O_3 layer. (c) Structural analysis of Ga_2O_3 films grown on five-layer MoS_2 using XRD 2θ-ω scans.

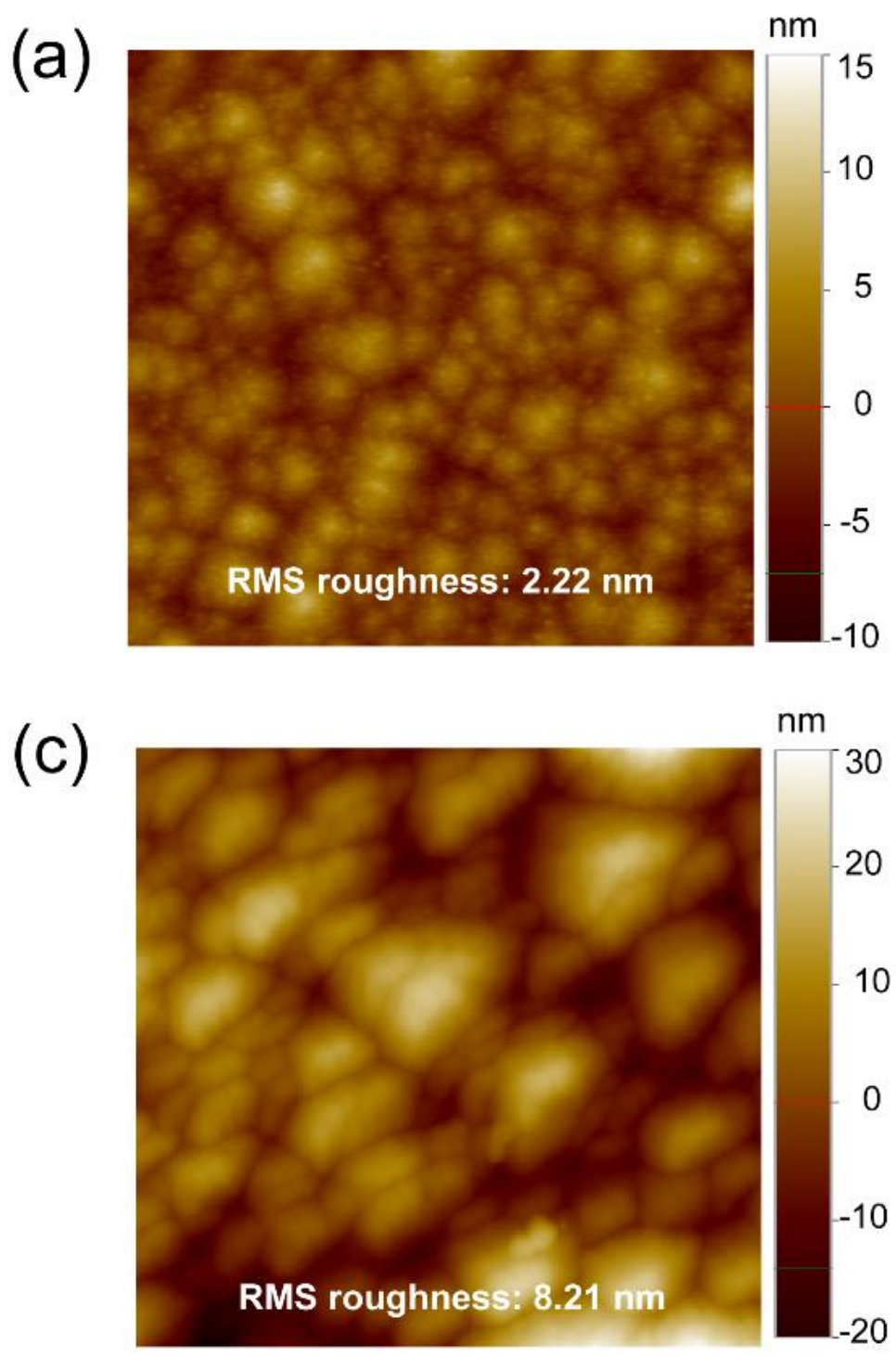

Supplementary Figure 3. Surface roughness and topography variations induced by MoS_2 templates. 5-by-5 μm^2 AFM images of Ga_2O_3 grown on (a) bare sapphire, (b) monolayer MoS_2, and (c) trilayer MoS_2. All films were grown for one hour.

Supplementary Table 1. Crystal quality of Ga_2O_3 measured by XRD rocking curves.

	Bare sapphire	Mono-layer MoS_2	Bi-layer MoS_2	Tri-layer MoS_2
(0006) reflection (arcsec)	36	45	1932	2690
$(10\bar{1}4)$ reflection (arcsec)	1100	1354	2237	3110

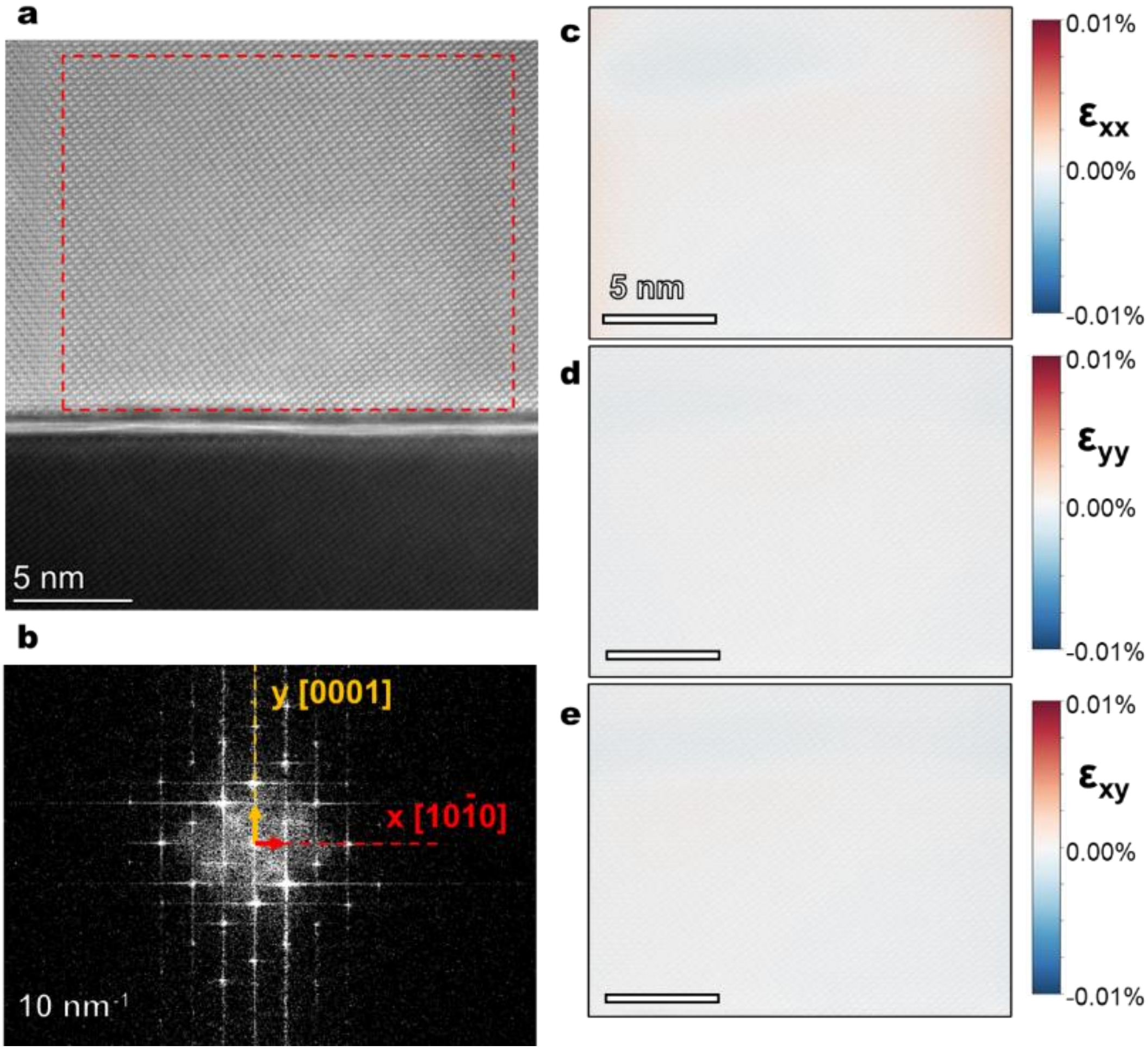

Supplementary Figure 4. Strain mapping of remote epitaxially grown α-Ga_2O_3 film on sapphire with a MoS_2 monolayer interlayer. (a) Cross-sectional HAADF-STEM image of the interface. The red dashed box indicates the region of the Ga_2O_3 film selected for strain mapping. (b) Corresponding fast Fourier transform (FFT) pattern, where x and y indicates the [10$\bar{1}$0] and [0001] crystallographic axes, respectively. (c) ε_{xx} (in-plane strain), (d) ε_{yy} (out-of-plane strain along c-axis), and (e) ε_{xy} (shear strain). The strain is calculated relative to the relaxed film region using geometric phase analysis (GPA). The color scale indicates strain values ranging from -0.01% to +0.01%.

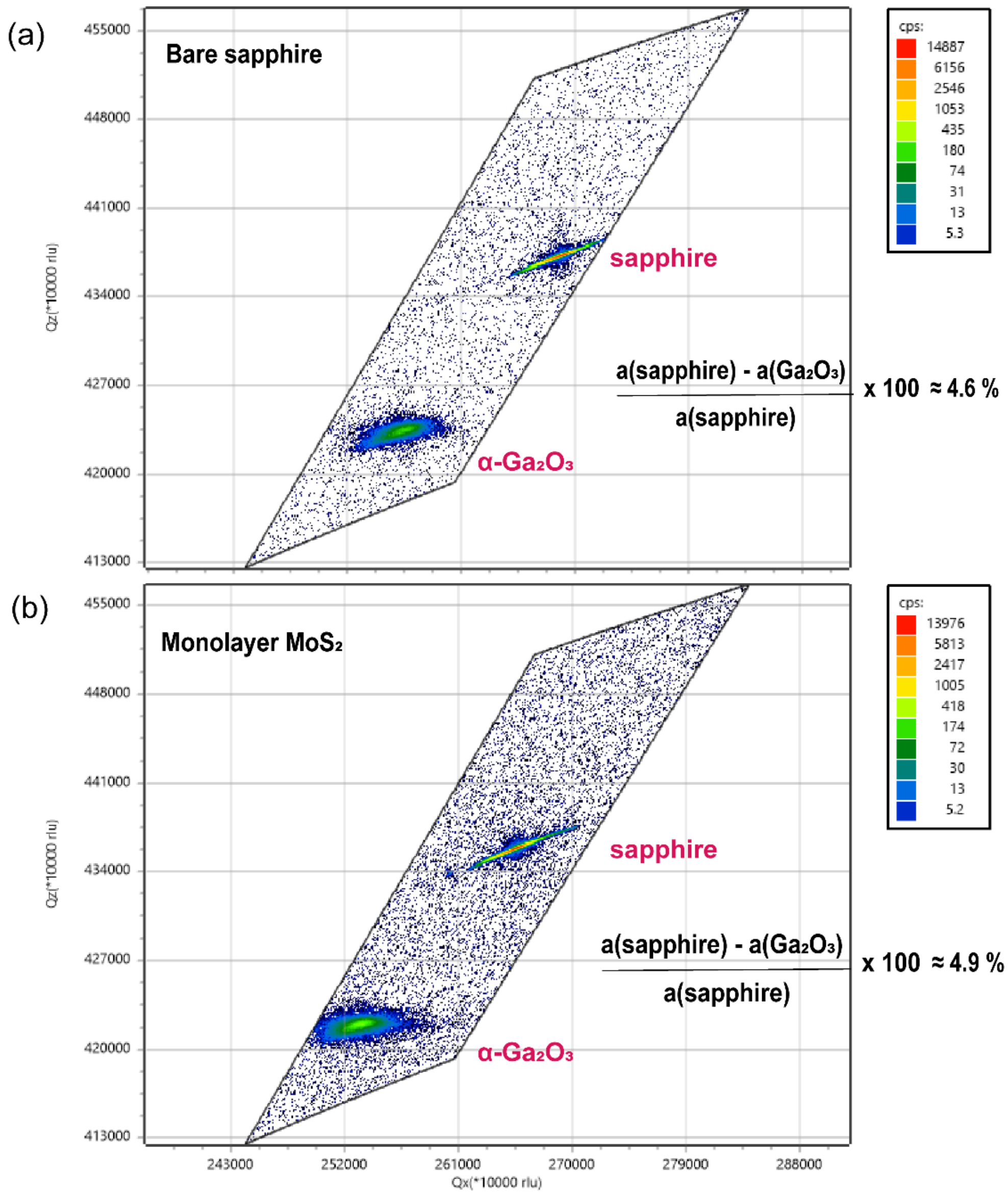

Supplementary Figure 5. Strain analysis via reciprocal space mapping (RSM) of α-Ga_2O_3. RSM of α-Ga_2O_3 on (a) bare sapphire and (b) monolayer MoS_2 on sapphire. Based on the reciprocal coordinate of Q_x, the in-plane lattice parameter, a, is obtained by using the equation, $Q_x = \frac{2\pi}{a}\sqrt{4/3(h^2 + k^2 + hk)}$, where h and k are the components of Miller indices.

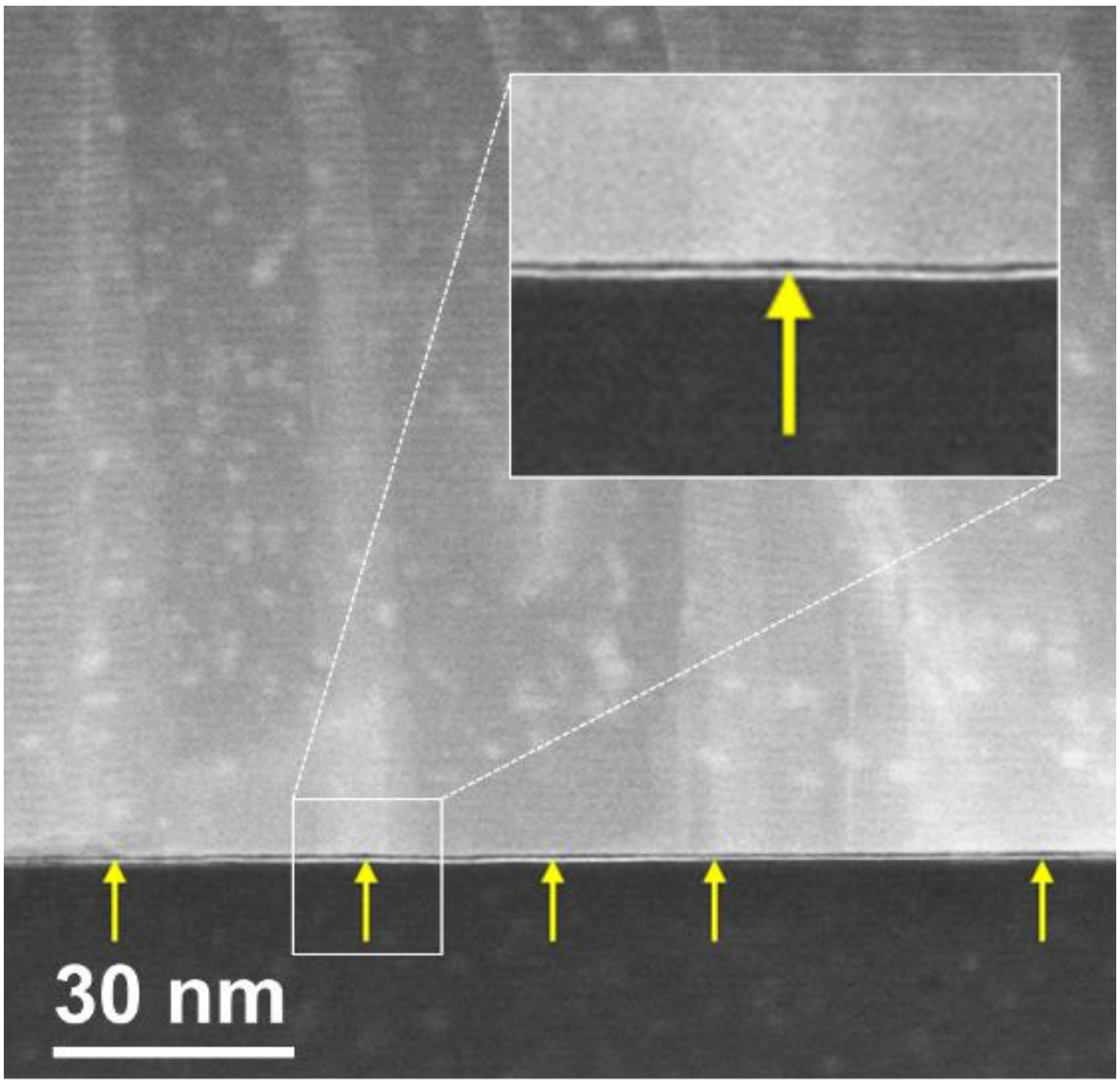

Supplementary Figure 6. Low-magnification cross-sectional analysis of threading dislocations. Representative low-magnification cross-sectional STEM image showing dislocations. Yellow arrows indicate threading dislocations originating from the interface and propagating toward the film surface.

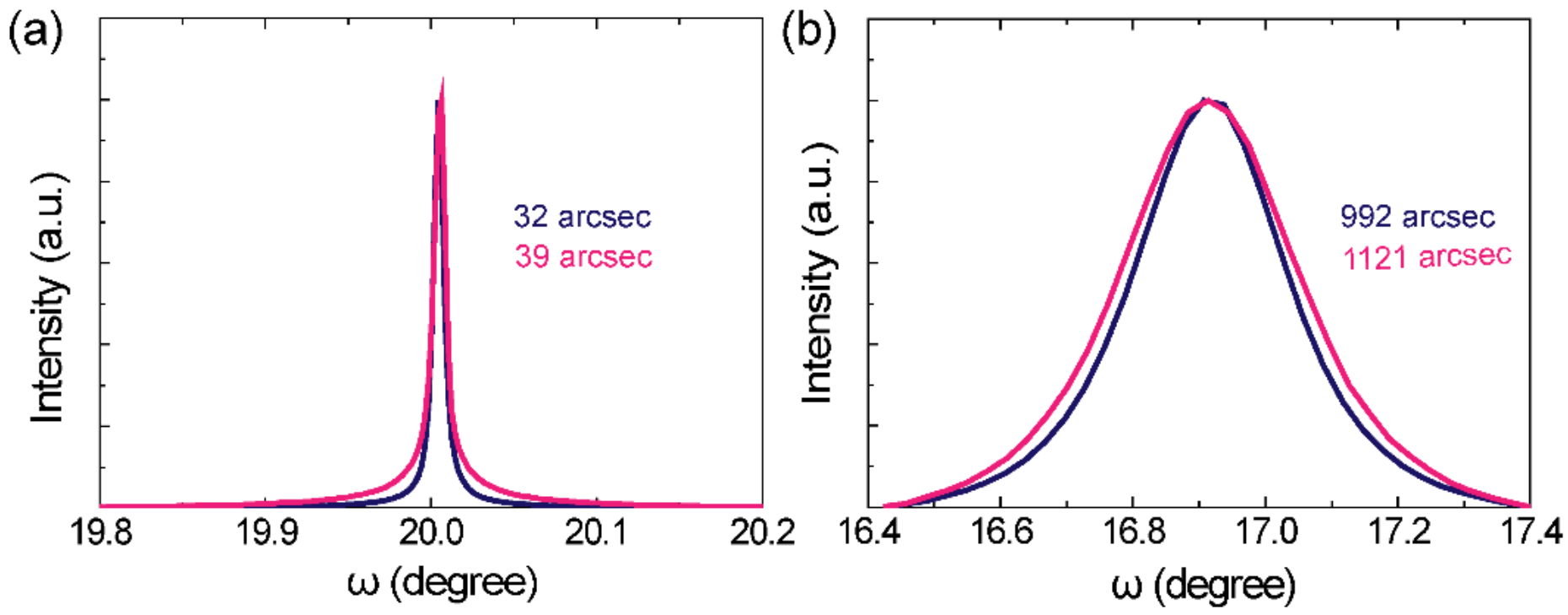

Supplementary Figure 7. Dependence of crystal quality of α-Ga_2O_3 on growth temperature. (a) On-axis (0006) reflection and (b) off-axis $(10\bar{1}\bar{4})$ reflection in rocking curve modes for the 1000-nm thick films grown at 470 °C.

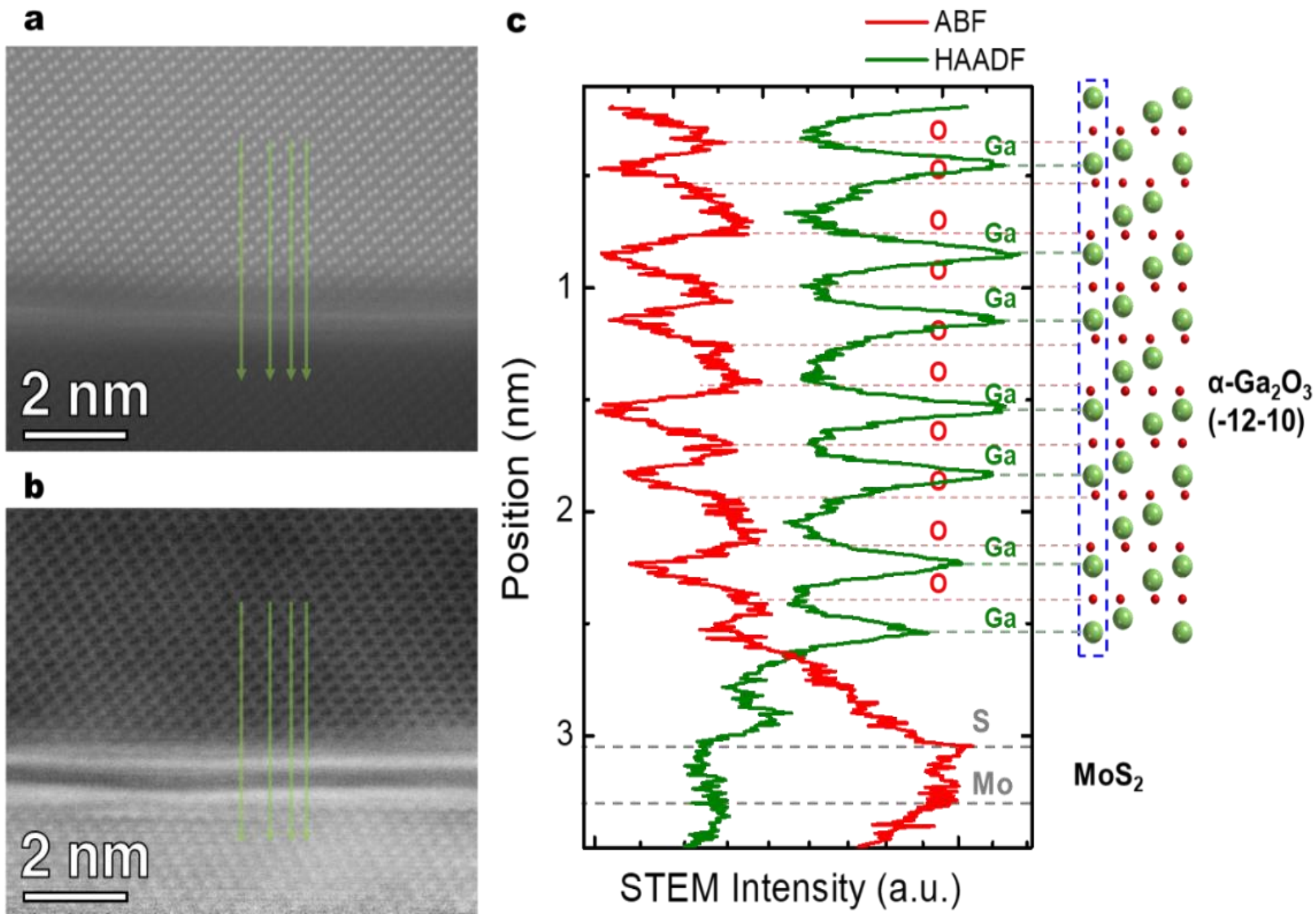

Supplementary Figure 8. Atomic-scale sub-lattice identification and intensity profiles at the interface. (a) HAADF and (b) ABF-STEM images of the $Ga_2O_3/MoS_2/Al_2O_3$ heterointerface acquired along the $[\bar{1}2\bar{1}0]$ zone axis. (c) Corresponding integrated intensity profiles for HAADF (green) and ABF (red) signals, with the atomic model shown on the right. The profiles were obtained by averaging intensities along multiple vertical paths (parallel to the c-axis), as indicated by the green arrows in (a) and (b). The blue dashed rectangle in the atomic model represents the lateral integration width used to isolate individual atomic columns. The combined HAADF and ABF-STEM images enable identification of both cation and anion sub-lattices, and the intensity profiles indicate that the first atomic layer above the MoS_2 corresponds to Ga cation columns, supporting a Ga-terminated interface.

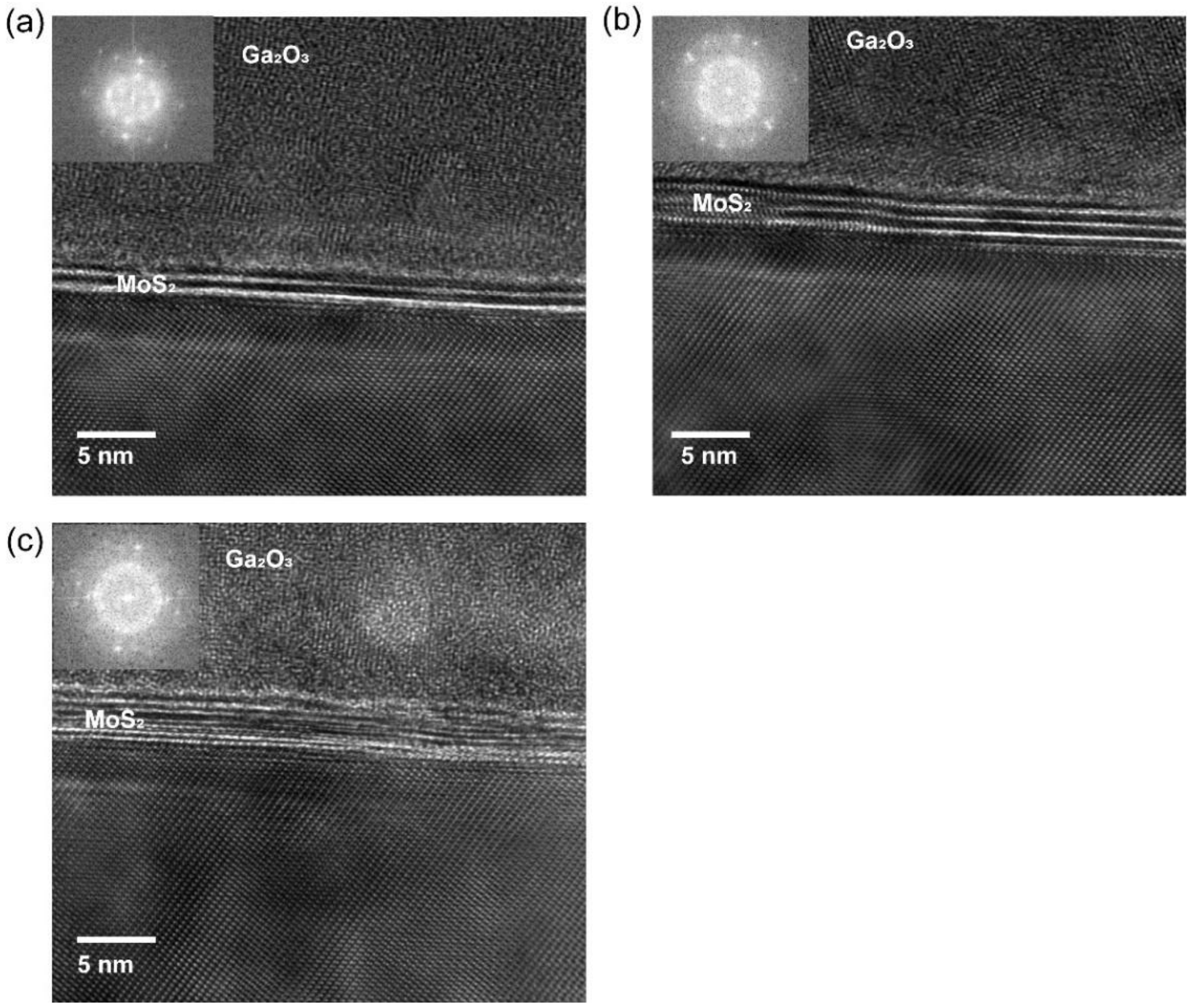

Supplementary Figure 9. High-resolution interface structures as a function of MoS_2 layer count. High-resolution cross-section TEM Images of Ga_2O_3 on (a) bilayer, (b) tri- and (c) four ~ five-layer MoS_2, with the insets showing the fast Fourier transform diffraction patterns of the Ga_2O_3 regions.

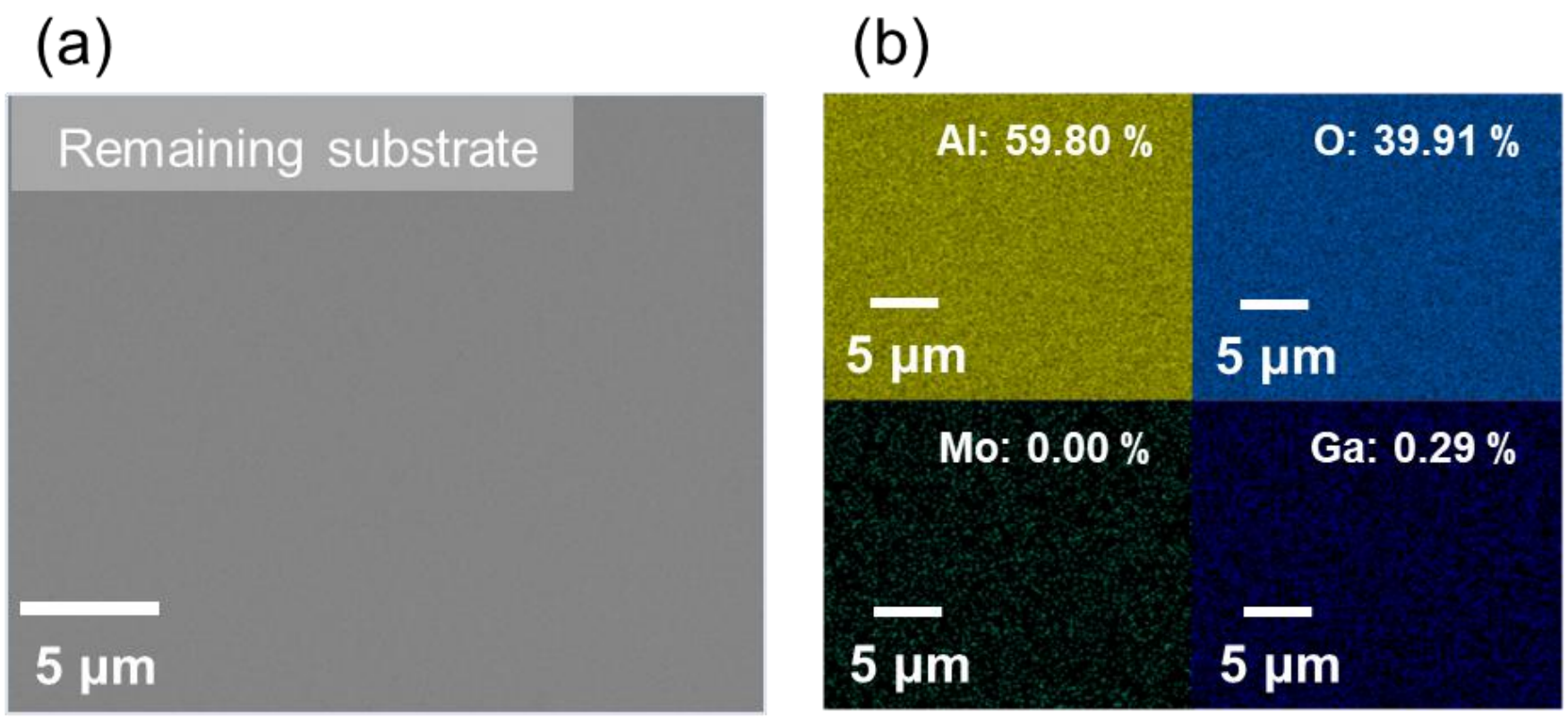

Supplementary Figure 10. Surface composition and SEM examination after mechanical exfoliation. (a) Top view SEM image of remaining substrate after exfoliation and (b) EDS analysis of the substrate surface.

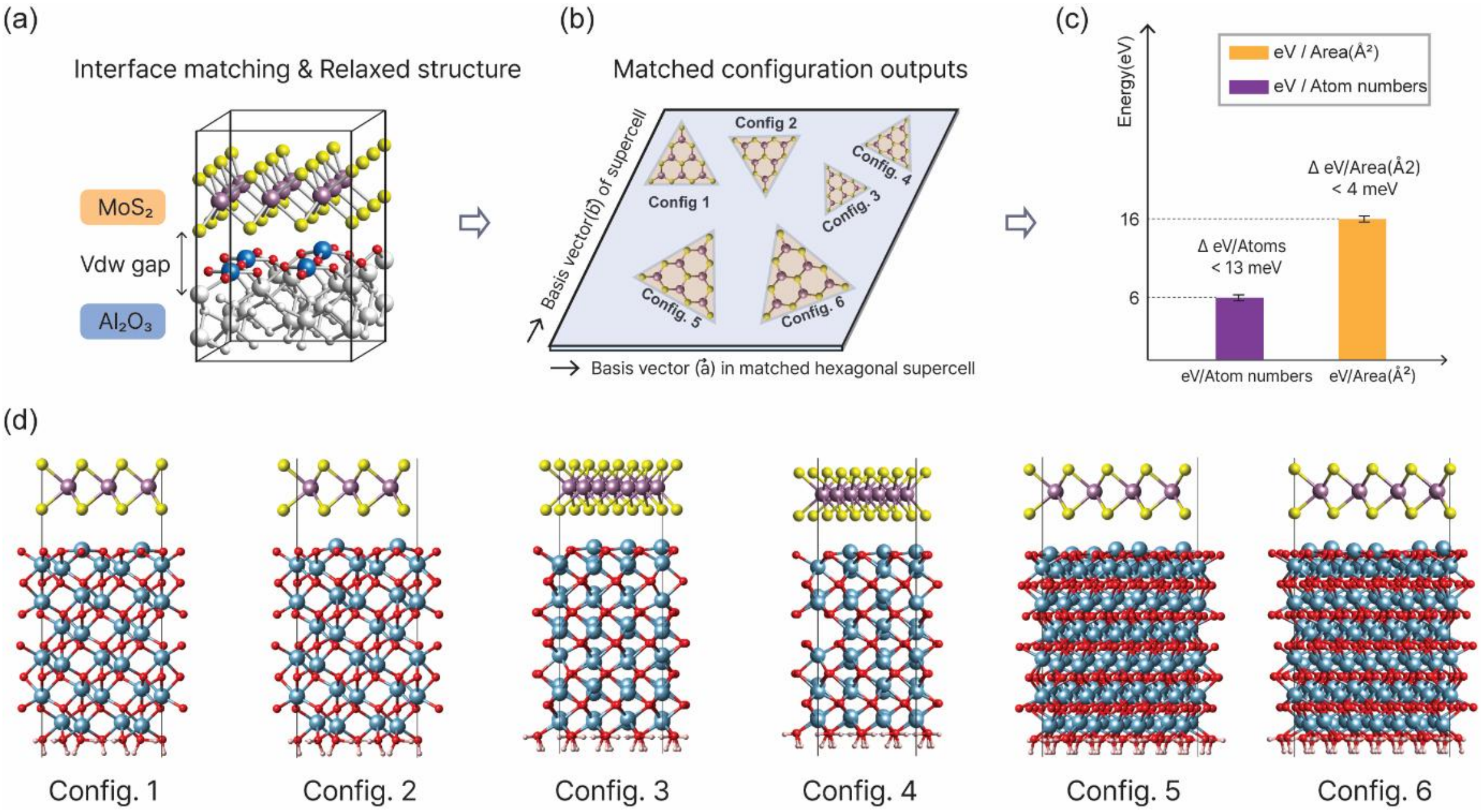

Supplementary Figure 11. Workflow and optimized energy states for DFT heterostructure models. Schematic overview illustrating the construction and geometric optimization process of the Al_2O_3/MoS_2 heterostructure interface using Density Functional Theory (DFT). (a) Heterostructure construction by systematically investigating possible combinations of Miller indices for Al_2O_3 substrate and MoS_2 layer. (b) Selected domain matching results achieved under a lattice vector mismatch constraint of less than 2 %. The final supercell is defined as a hexagonal unit cell with basis vectors $\vec{a}$ and $\vec{b}$, aligned parallel to specific linear combinations of primitive lattice vectors from the Al_2O_3 substrate and MoS_2 layer. (c) Bar chart depicting relaxation energies(ΔE) for various matched MoS_2/Al_2O_3 heterostructure supercells. Orange bars represent ΔE normalized per unit interface area (eV Å^{-2}), while purple bars denote ΔE normalized per atom (eV/atom). The maximum variation in these relaxation energies is minimal, remaining below 4 meV Å^{-2} and 13 meV per atom. (d) Relaxed atomic configurations resulting from structural optimization via DFT calculations, illustrating stable interface geometries.

Supplementary Table 2. Linear combinations of primitive lattice vectors for Al_2O_3 substrate and MoS_2 layer studied in this study. The table lists the selected combinations of supercell basis vectors ($\vec{a}$ and $\vec{b}$) defining the interface orientations of heterostructure models 1 to 6.

		Model 1	Model 2	Model 3	Model 4	Model 5	Model 6
Vector $\vec{a}$ (Al_2O_3 || MoS_2)	Al_2O_3	$[1\ \bar{1}\ 0]$	$[1\ \bar{1}\ 0]$	2[1 0 0]	2[1 0 0]	[3 1 0]	[3 1 0]
	MoS_2	$[2\ \bar{1}\ 0]$	$[2\ \bar{1}\ 0]$	$3[0\ \bar{1}\ 0]$	$3[0\ \bar{1}\ 0]$	$4[0\ \bar{1}\ 0]$	$4[0\ \bar{1}\ 0]$
Vector $\vec{b}$ (Al_2O_3 || MoS_2)	Al_2O_3	[2 1 0]	[2 1 0]	2[1 1 0]	2[1 1 0]	[2 3 0]	[2 3 0]
	MoS_2	[3 2 0]	[3 2 0]	3[1 0 0]	3[1 0 0]	4[1 0 0]	4[1 0 0]

Supplementary Table 3. Calculated areas, percentages of lattice mismatch (strain), energies per atom, and total energies per unit area of the optimized Al_2O_3/MoS_2 heterostructure models (Models 1–6). These structural and energetic parameters were obtained through geometric optimization based on DFT calculations.

Features	Model 1	Model 2	Model 3	Model 4	Model 5	Model 6
Area (Å^2)	79.980	79.980	59.985	59.985	139.965	139.965
Strain (%)	0.563	0.563	1.250	1.250	0.226	0.226
Energy/atom (eV)	6.9047	6.9055	6.9084	6.9082	6.9069	6.9072
Energy/area (eV/Å^2)	16.1439	16.1455	16.2389	16.2384	16.1858	16.1866

Supplementary Table 4. Comparison of relaxed atomic structures of AlO_3/MoS_2 heterojunctions with Half-Al, Al, and O terminations, illustrating structural differences affecting stability and electronic properties. The table presents detailed data on interlayer distances, per-atom energies, and Bader charge differences for the bottom S atoms for the Half-Al, Al, and O-terminated interfaces.

Features	Half-Al termination	Al-termination	O-termination
Relaxed atomic structure	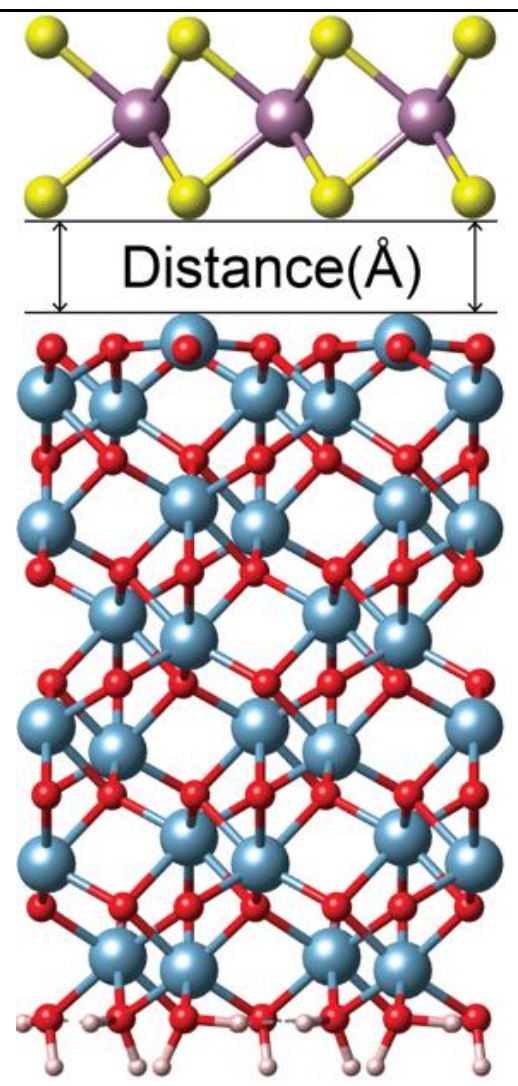	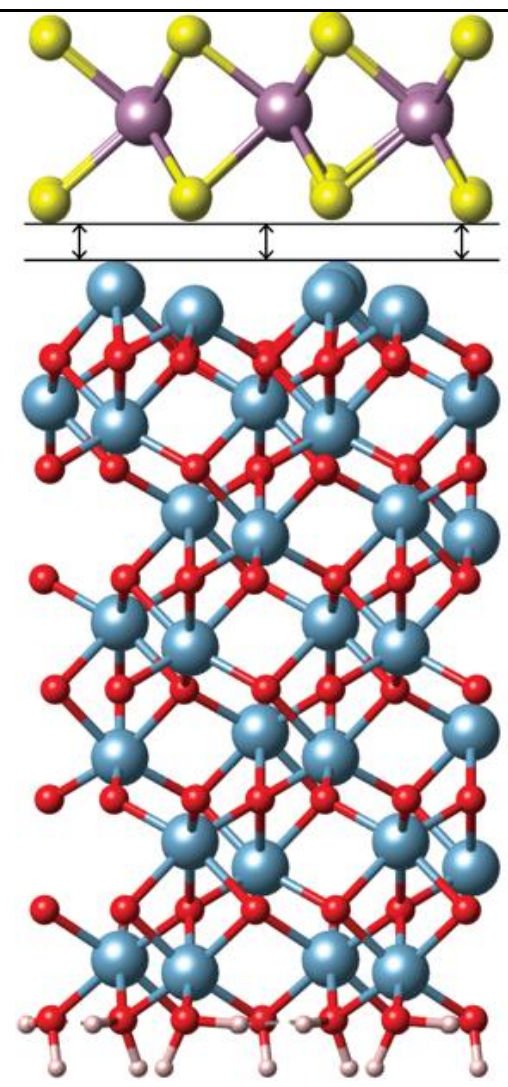	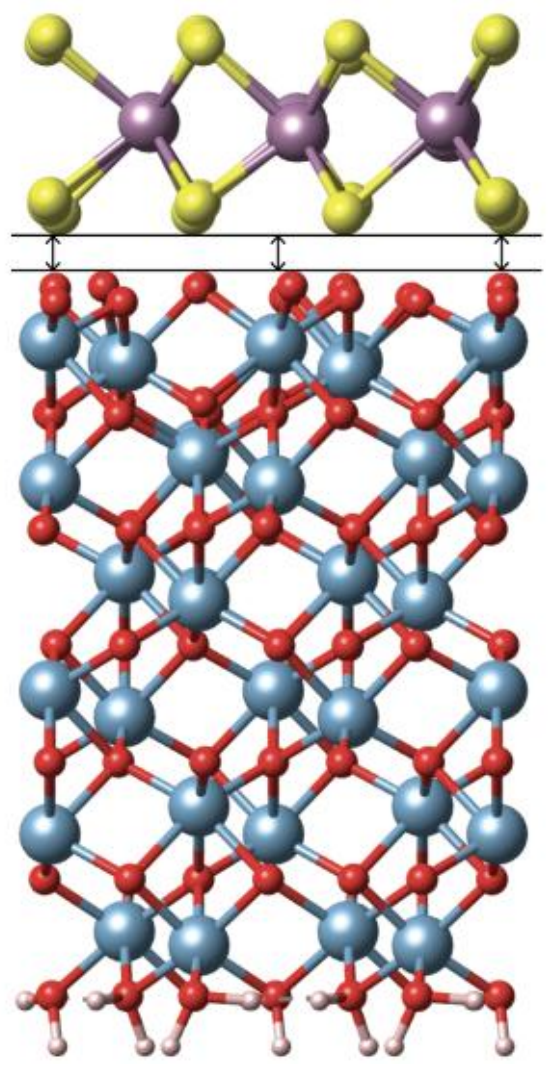
Interlayer distance (Å)	2.81 Å	1.82 Å	1.37 Å
Energy per atom	-8.069 eV/atom	-7.862 eV/atom	-7.962 eV/atom
Δ Bader charge of bottom S atoms (e)	0.036 e	0.397 e	0.649 e

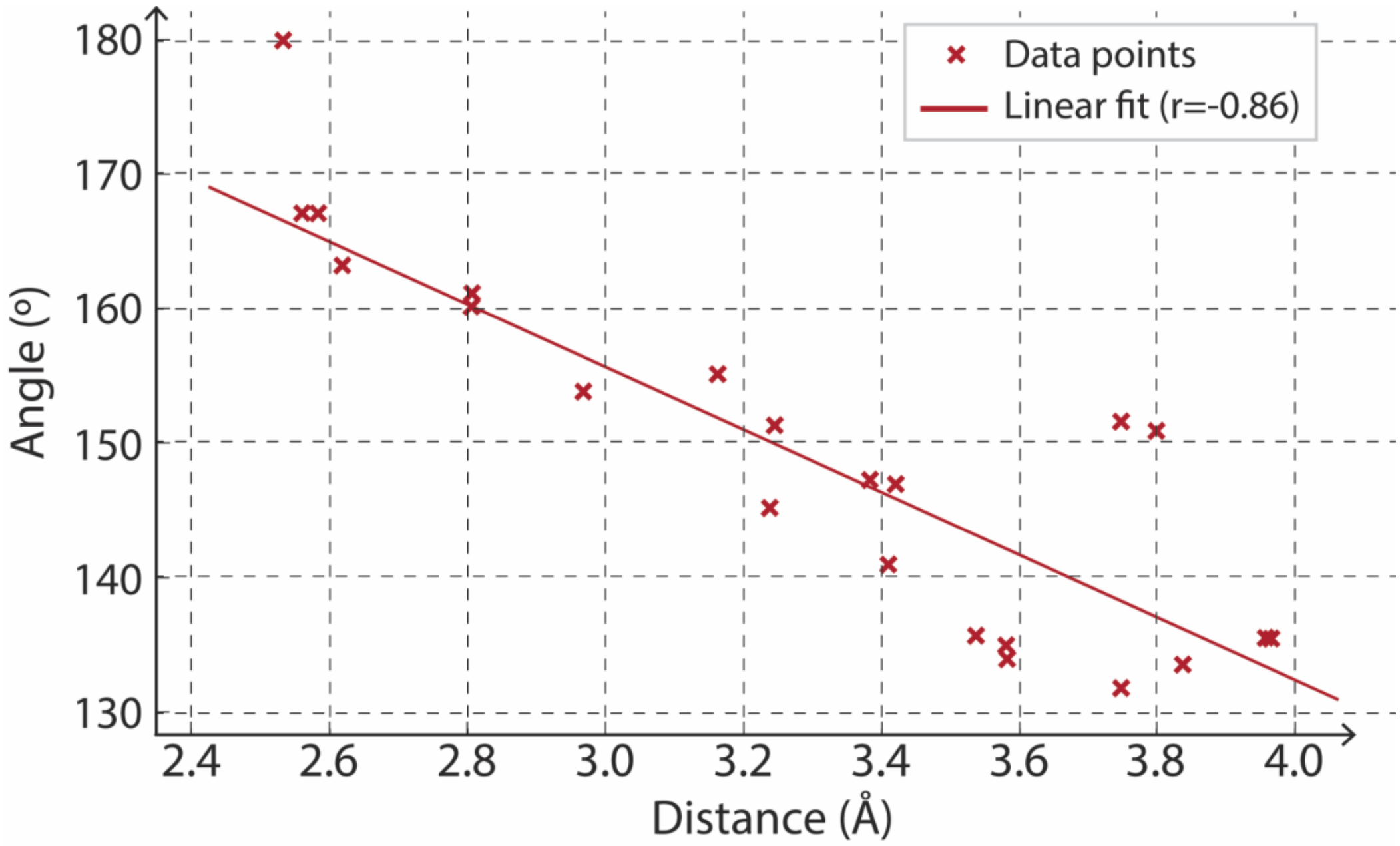

Supplementary Figure 12. **Geometrical correlation between interatomic distance and interfacial bond angle.** Correlation between interatomic distance (Al_{top}-S_{bott}) and bond angle ($\angle Al_{top}$-S_{bott}-S_{top}). Red crosses represent 65 individual (distance, angle) pairs sampled from distinct S_{top} atomic sites; due to symmetry and slight overlaps in distance-angle values, fewer distinct points (22) are visually resolved in the plot. The red solid line is a least-squares linear regression (slope = –18.4 ° $Å^{-1}$; intercept = 214 °) that captures a strong inverse linear trend (Pearson correlation coefficient r = –0.86, $R^2 \approx 0.74$). The result indicates that as the A_{top}-S_{bott} interatomic distance decreases, the associated Al_{top}-S_{bott}-S_{top} bond angle increases towards 180°.

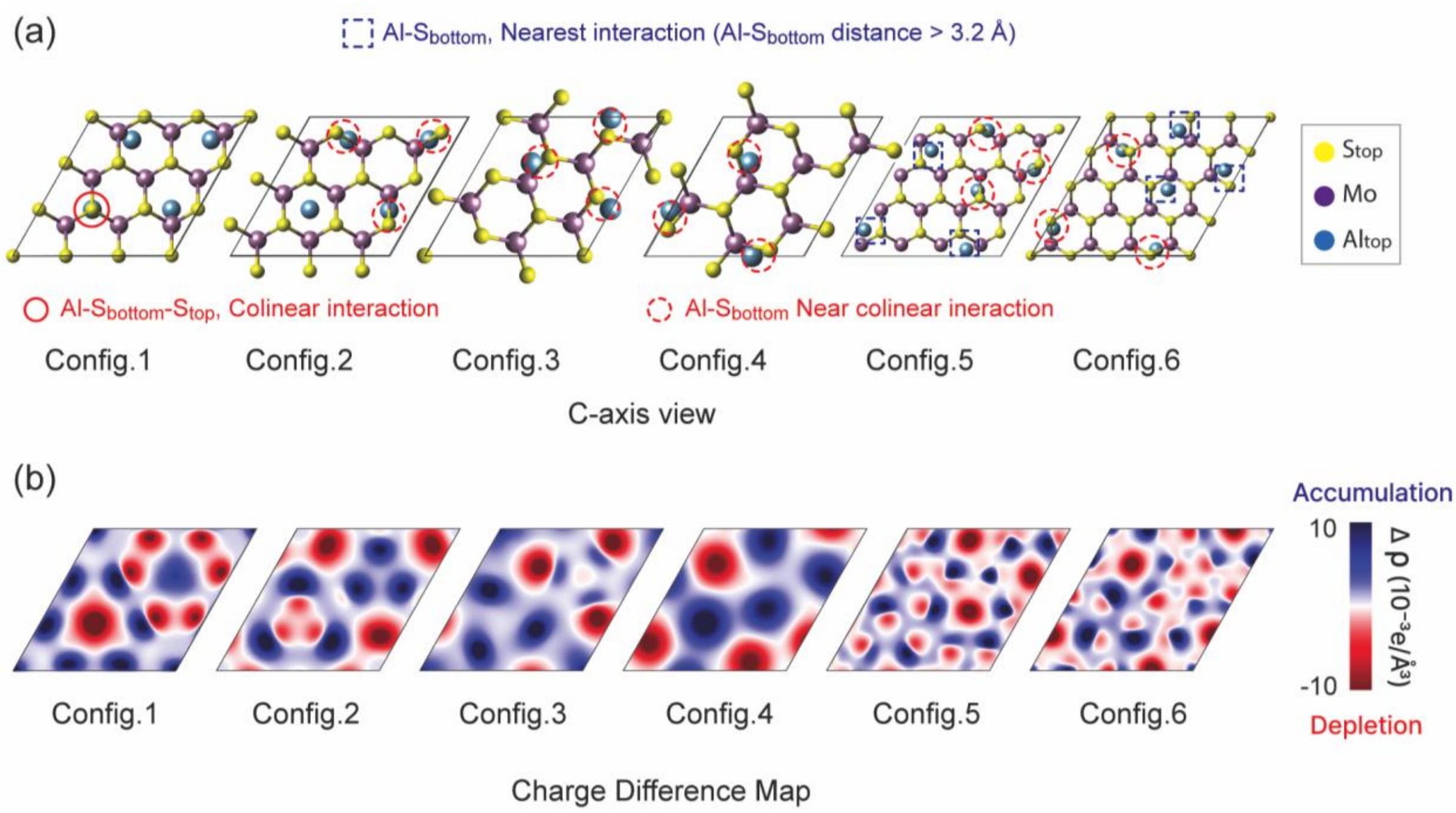

Supplementary Figure 13. Configuration-dependent charge density difference at the Al_2O_3/MoS_2 interface. Calculated interfacial atomic configurations and charge redistribution at the Al_2O_3/MoS_2 heterostructure. (a) C-axis views of six atomic configurations (Config 1-6) at the Al_2O_3–MoS_2 interface. Each configuration is distinguished by the type of Al-S_{bottom}-S_{top} interaction. Key interaction types are as follows: (1) Al-S_{bottom}-S_{top} collinear interactions: Defined where the minimum Al-S_{bottom}-S_{top} distance corresponds to the Van der Waals (VdW) gap (solid red circles). (2) Al-S_{bottom} nearest interactions: Characterized by an Al-S_{bottom} distance > VdW gap (dashed red circles). (3) Al-S_{bottom} nearest interactions: Occurring where the Al-S distance > 3.2 Å (dashed green rectangles). (b) Corresponding charge difference ($\Delta\rho$) maps for Config 1-6, clearly illustrating electron accumulation (red regions) and depletion (blue regions) at the interface.

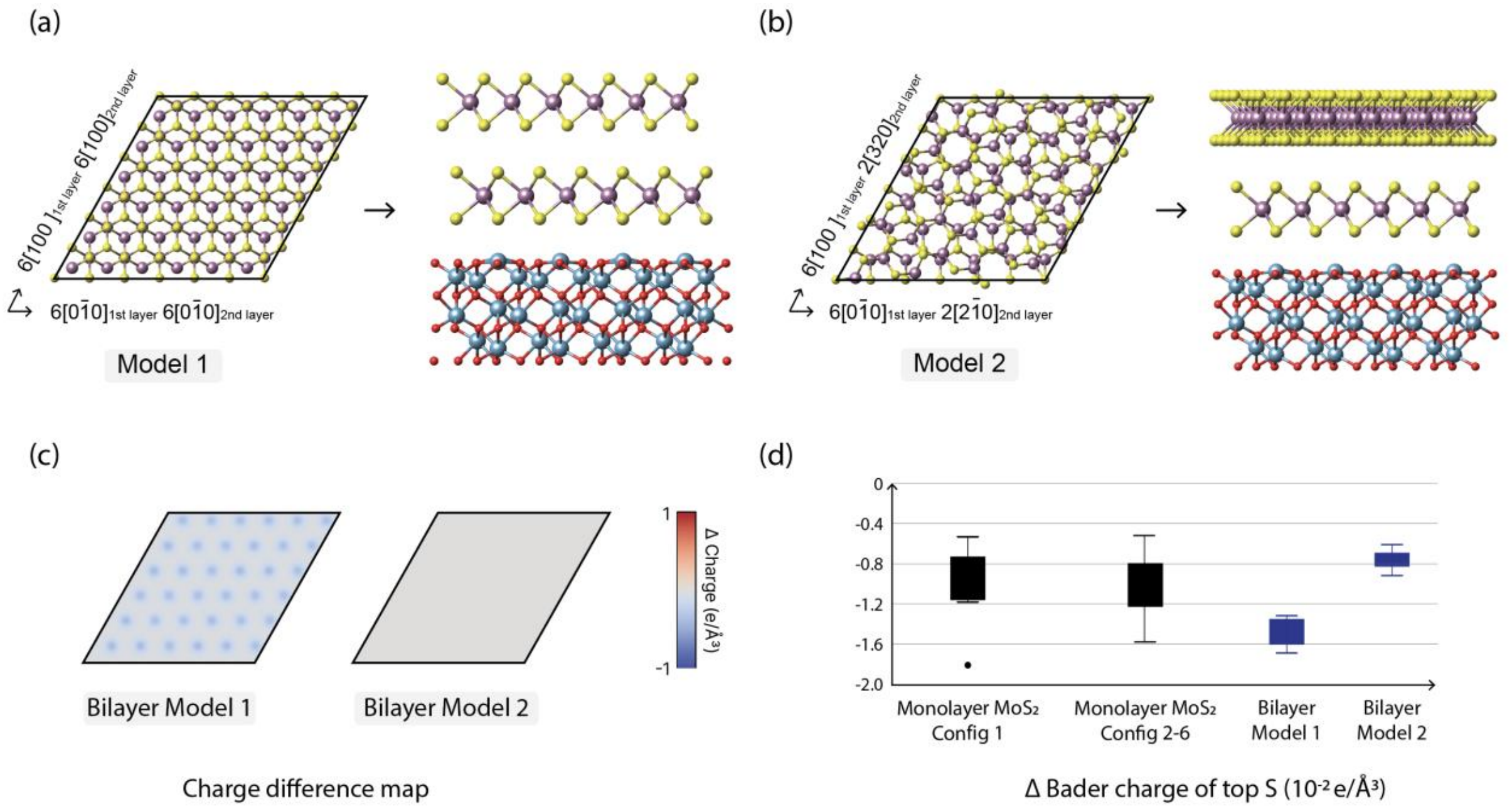

Supplementary Figure 14. Charge transfer analysis for the Al_2O_3/Bilayer MoS_2 hetero-structure. (a) Schematic representation of the Al_2O_3/bilayer MoS_2 (model 1). An optimized, relaxed bilayer structure comprises a first layer in configuration 1 (as defined in the main text) and a second layer in configuration 2 (as described in the main text). (b) Schematic representation of the Al_2O_3/bilayer MoS_2 (model 2). A bilayer structure consists of a first layer in configuration 1 and a second layer in configuration 3. (c) Charge difference maps for the bilayer model 1 and bilayer model 2, illustrating spatial redistributions of electronic charge at the top sulfur atoms, providing insight into the interfacial charge transfer. (d) A bar graph comparing the Bader charges of the top sulfur atoms (in units of 10^{12} e/Å^2) across various configurations: monolayer MoS_2 (config. 1), monolayer MoS_2 (Configs. 2–6), bilayer model 1, and bilayer model 2, with error bars included to indicate the variability in calculations. In the case of the config. 1 monolayer, the presence of an outlier value in the charge transfer mediated by the sulfur atom, which is influenced by the substrate's crystallographic information, is observed; however, this effect is reduced and more uniformly distributed across the remaining configurations. Furthermore, the relatively small variation in charge transfer within the bilayer suggests a low degree of charge transfer localized to specific atoms.

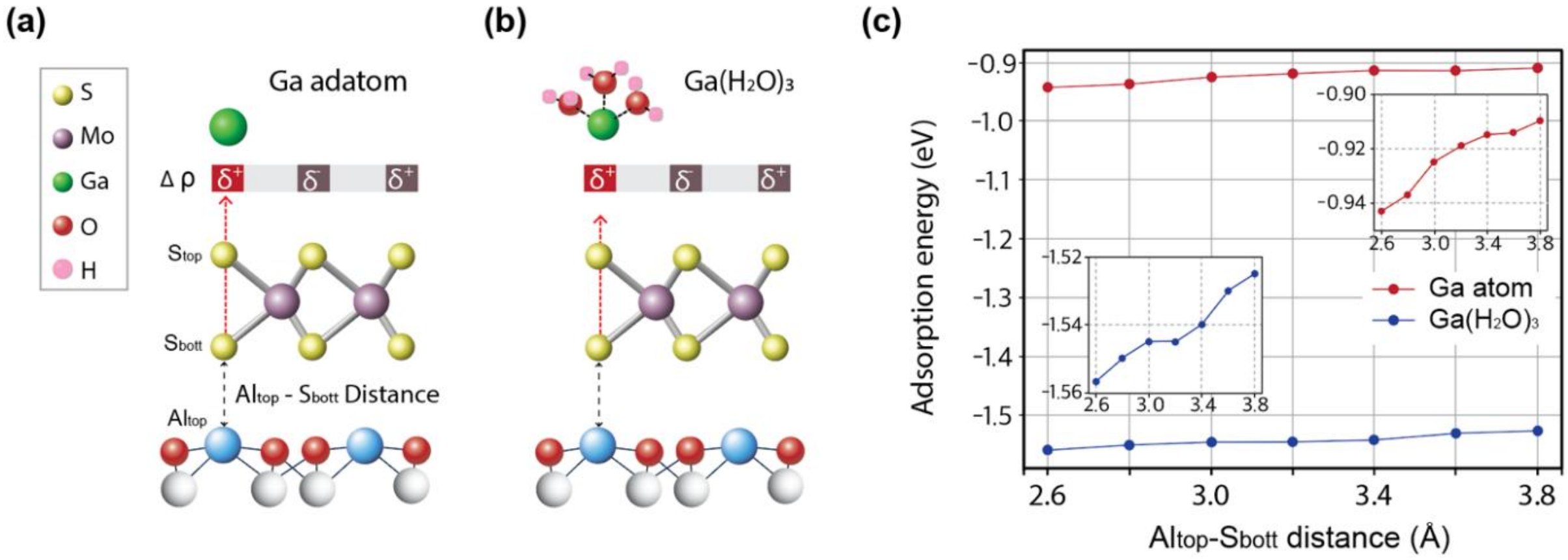

Supplementary Figure 15. Adsorption energies of Ga species on MoS_2 modulated by charge redistribution induced by Al_2O_3–MoS_2 interactions. (a) Schematic illustration of a Ga adatom adsorbed on a charge-depletion region, located above an S_{top} site of MoS_2 on the sapphire substrate. (b) Schematic illustration of a $Ga(H_2O)_3$ complex adsorbed at the identical site. This structure model the initial adsorbing species in the mist CVD process (*45*). (c) Calculated adsorption energies for the Ga adatom (blue line) and the $Ga(H_2O)_3$ complex (red line) as a function of the Al_{top}-S_{bott} atomic distance. These results show that both the Ga adatom and Ga complex species adsorb more strongly at charge-depleted sites, a trend consistent with our calculations (see Figure 5d). Collectively, these results demonstrate that long-range electrostatic potentials originating from the sapphire (Al_{top})–MoS_2 (S_{bott}) interaction spatially modulate the charge distribution on the MoS_2 surface, ultimately leading to site-selective adsorption of Ga species.

Supplementary Note 1. DFT simulations for the Al_2O_3/MoS_2 interface modeling

In this work, density functional theory (DFT) calculations were performed to elucidate the underlying mechanism enabling freestanding single-crystal α-Ga_2O_3 growth via a polycrystalline MoS_2 interlayer. To investigate the electronic charge distribution of MoS_2 interlayer influenced by the polar Al_2O_3(0001) substrate, we identified Al_2O_3/MoS_2 hetero-strucuture by interface domain matching algorithms (*50*). The preliminary Al_2O_3/MoS_2 interface modeling process are presented in Supplementary Fig. 11(a). To search for an ideal hetero-interface, various combinations of substrate and film Miller indices were considered. From this method, optimized domain-matched configurations with the lowest mismatch strain, supercell area, and lattice vector length were identified (see Supplementary Table 2 and 3). Consequently, six domain-matched heterostructures between Al_2O_3 and MoS_2 surfaces were identified, each exhibiting a lattice mismatch of less than 2%. Supplementary Fig. 12(b) presents a comparison of the total system energies obtained after geometric relaxation by DFT calculation, with the energy deviation for each configuration being less than 13 meV per atom and less than 4 meV per unit area. These small energy differences indicate that several crystallographic orientations are equivalently favored, which is consistent with the synthesized polycrystalline monolayer of MoS_2 (*51*).

Although the energy differences among various Al_2O_3/MoS_2 interface configurations are minimal, the detailed nature of this interface is crucial for developing an accurate model to assess the impact of the polar substrate. In particular, whether the Al_2O_3 surface is terminated by aluminum or oxygen atoms strongly affects bonding and charge transfer at the heterointerface (*52*). Based on the early research conducted by Soares et al. (*53*) and Kurita et al. (*54*), we have classified three surface-termination states distinguished by unsaturated ionic charges: Al-terminated, O-terminated, and half-Al terminated ones. As shown in Supplementary Table 4, aluminum atoms at Al-terminated, half-Al terminated Al_2O_3 surfaces exhibit weak charge transfer (0.649 e) with sulfur atoms in MoS_2 layer, whereas oxygen atoms at O-terminated surfaces exhibit stronger charge transfers (0.036 e) with sulfur. On the other hand, the half-Al terminated surface not only represents the most stable state in terms of the system energy on a per-atom and per-unit area, but it is also marked by minimized charge transfer with interlayer ions. Consequently, the half-Al terminated Al_2O_3 surface prevents the direct covalent-bond formation with the MoS_2 layer, which is not the case for the other surface

states.

Epitaxial growth of Ga_2O_3 via mist CVD typically proceeds through a ligand-exchange pathway, in which the Ga(III) precursor reacts with surface hydroxyl (–OH) groups, forming Ga–O bonds with lattice oxygen atoms (*45*). However, in our experiments, successful Ga_2O_3 film growth was observed on the MoS_2 surface. This observation suggests that nucleation may not have been governed by conventional chemical bonding through ligand exchange. Instead, it is likely facilitated by a site-specific adsorption environment (i.e., charge-depleted sites) for Ga precursors, the origin of which is proposed to arise from long-range interactions between the Al_2O_3 substrate and the MoS_2 layer. To further support this hypothesis, we calculated the adsorption energies of hydrated $Ga(H_2O)_3$ species in addition to those of the Ga adatom. The adsorption energies were evaluated above the S_{top} site as a function of the Al_{top}–S_{bott} distance (see Supplementary Figure 14), showing a similar trend to that observed in the Ga adatom calculations.

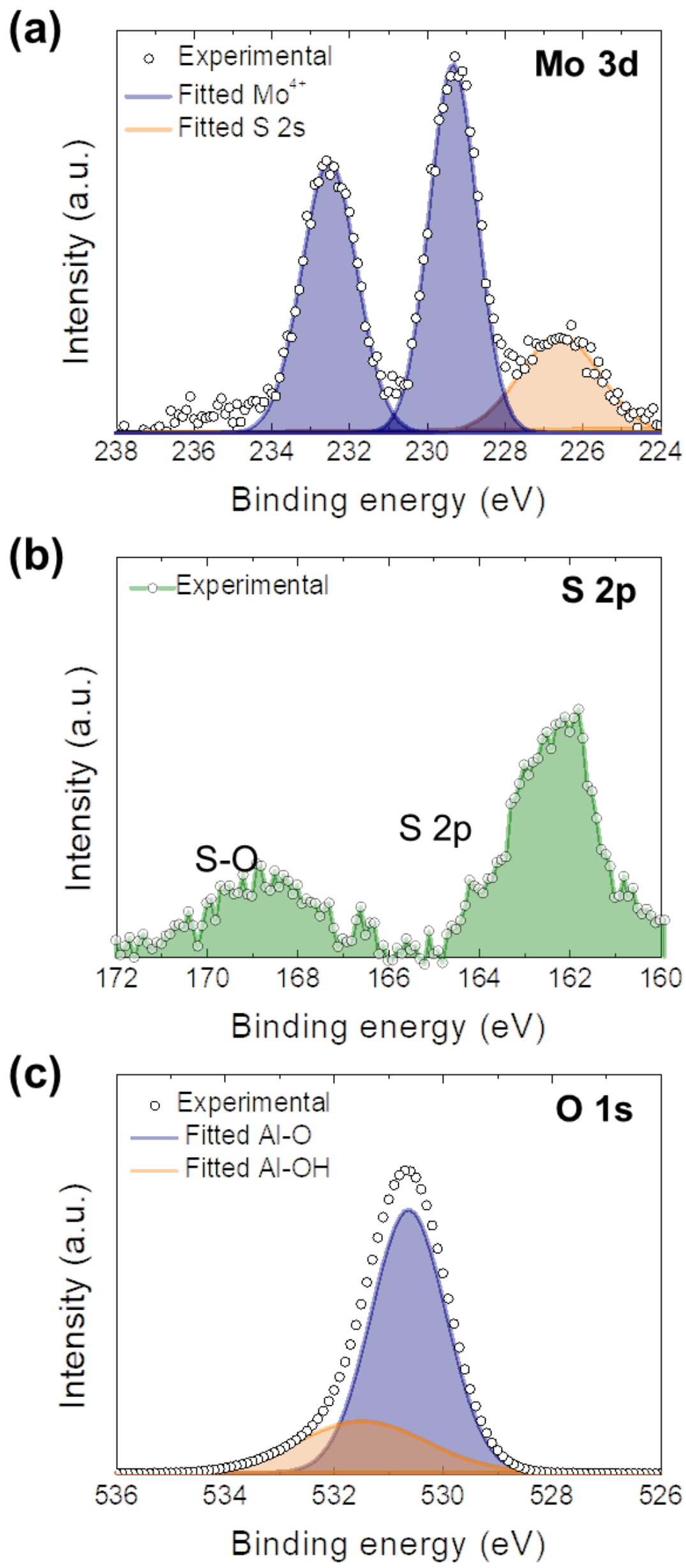

Supplementary Figure 16. X-ray photoemission spectroscopy (XPS) measurement on monolayer MoS_2/sapphire. Show are the spectra for (a) Mo 3d and S 2s corresponding to stoichiometric MoS_2, (b) S 2p corresponding to stoichiometric MoS_2 and S-O bonding, and (c) O 1s primarily from the sapphire substrate. Experimental data are displayed as dotted lines, and fitted data are represented as filled curves. The feature at ~234 - 237 eV in (a) and the S 2p signal in (b) show insufficient signal-to-noise ratio for reliable peak deconvolution.

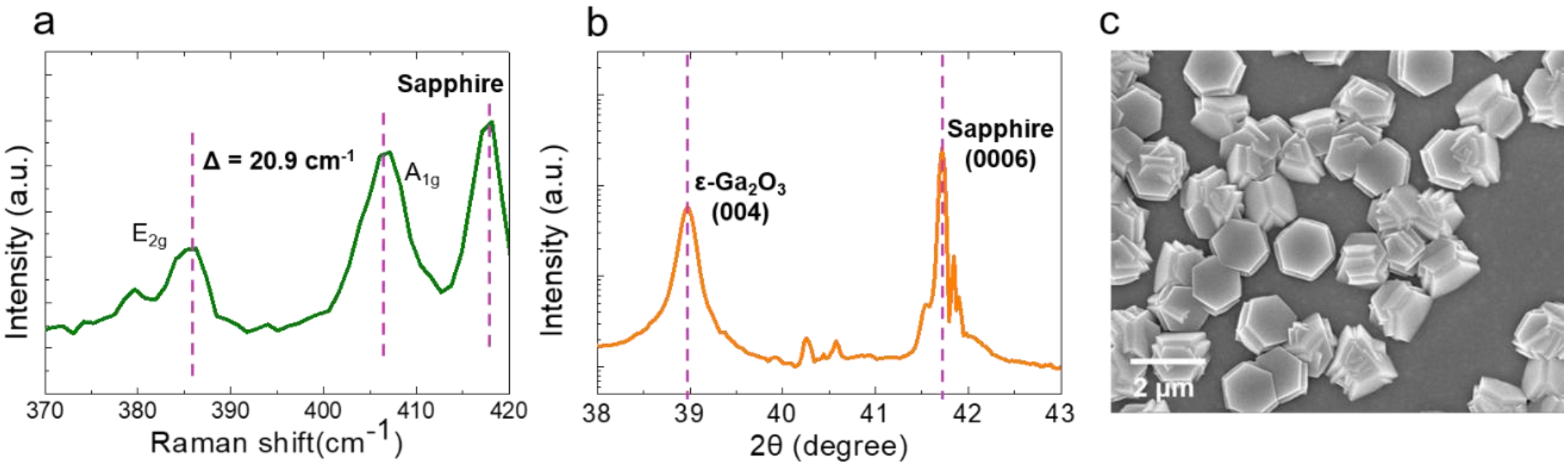

Supplementary Figure 17. Effects of surface oxygen plasma treatment on crystalline phase and morphology. (a) Raman spectrum of MoS_2 on O_2-plasma-treated sapphire, confirming monolayer thickness with a peak separation of $\Delta E \approx 20.9\ cm^{-1}$, (b) XRD 2θ–ω scan of Ga_2O_3 grown on the MoS_2 template, indicating the formation of the ε-phase instead of the α-phase observed under remote epitaxy conditions, and (c) SEM image showing the surface morphology of the Ga_2O_3 film, exhibiting a hazy appearance with hexagonal features similar to those observed on thicker MoS_2 layers.

Supplementary Note 2. XPS analysis of the Al_2O_3/MoS_2 interface

When MoS_2 is synthesized by sulfurizing MoO_3 powder, previous studies have shown that annealing the sapphire substrate under H_2 flow at temperatures above 500 °C effectively removes surface-adsorbed –OH groups, yielding an Al-half-terminated surface (*55*). On such pretreated sapphire substrates, in-situ growth of MoS_2 leads to highly selective, well-aligned single-crystalline 2D MoS_2 films. In addition, it was also reported that thermal processing at 900 °C alone can achieve c-plane sapphire surface with an Al-half-terminated state (*55*).

In our solution-based thermolysis, MoS_2 is synthesized from a precursor solution through a coating process carried out in ambient air; consequently, in-situ H_2 pretreatment of the sapphire surface is not feasible. This suggests that a residual density of S-O bonds could be present, possibly interfering with the remote epitaxy process.

X-ray photoelectron spectroscopy (XPS) analysis (Supplementary Fig. 16) of our standard monolayer MoS_2 template confirms that the Mo 3d and S 2p spectra are dominated by features associated with Mo–S bonding, indicating that it is the predominant chemical state. A minor feature observed in the S 2p spectrum at ~168–169 eV is attributed to oxidized sulfur

species (S–O bonding). The relatively low intensity of this component indicates that these S-O bonds represent only a minor contribution compared to the primary Mo–S bonding states. Additionally, the O 1s spectrum is primarily composed of contributions from the sapphire surface lattice oxygen and hydroxyl-related species. All peak assignments were made based on several reference spectra (*55-59*).

In addition to XPS analysis, a control experiment was conducted where the sapphire surface was treated with O_2 plasma. While a standard monolayer MoS_2 was synthesized (Supplementary Fig. 17(a)), subsequent Ga_2O_3 growth failed to yield the expected α-phase. Instead, XRD identified the ε-Ga_2O_3 phase (Supplementary Fig. 17(b)), and the surface morphology (Supplementary Fig. 17(c)) exhibited hexagonal features characteristic of ε-Ga_2O_3 typically on thicker MoS_2 templates (see Fig. 2(f) in the manuscript for comparison). These results suggest that a high density of S-O bonds at the MoS_2/sapphire interface disrupts the successful remote nucleation of α-Ga_2O_3.

REFERENCES

1. Y. Kim, S. S. Cruz, K. Lee, B. O. Alawode, C. Choi, Y. Song, J. M. Johnson, C. Heidelberger, W. Kong, S. Choi, K. Qiao, I. Almansouri, E. A. Fitzgerald, J. Kong, A. M. Kolpak, J. Hwang, J. Kim, Remote epitaxy through graphene enables two-dimensional material-based layer transfer. *Nature* **544**, 340–343 (2017).

2. B.-I. Park, J. Kim, K. Lu, X. Zhang, S. Lee, J. M. Suh, D.-H. Kim, H. Kim, J. Kim, Remote Epitaxy: Fundamentals, Challenges, and Opportunities. *Nano Lett.* **24**, 2939–2952 (2024).

3. H. S. Kum, H. Lee, S. Kim, S. Lindemann, W. Kong, K. Qiao, P. Chen, J. Irwin, J. H. Lee, S. Xie, S. Subramanian, J. Shim, S.-H. Bae, C. Choi, L. Ranno, S. Seo, S. Lee, J. Bauer, H. Li, K. Lee, J. A. Robinson, C. A. Ross, D. G. Schlom, M. S. Rzchowski, C.-B. Eom, J. Kim, Heterogeneous integration of single-crystalline complex-oxide membranes. *Nature* **578**, 75–81 (2020).

4. M. Park, T. Maekawa, K. Hwang, J. Cable, W. Noriyuki, K. Choi, Y.-K. Noh, Y. Oh, Y. Baek, K. Lee, Remote epitaxy and freestanding wide bandgap semiconductor membrane technology. *Nat. Rev. Electr. Eng.* **1**, 680–689 (2024).

5. J. Shin, H. Kim, S. Sundaram, J. Jeong, B.-I. Park, C. S. Chang, J. Choi, T. Kim, M. Saravanapavanantham, K. Lu, S. Kim, J. M. Suh, K. S. Kim, M.-K. Song, Y. Liu, K. Qiao, J. H. Kim, Y. Kim, J.-H. Kang, J. Kim, D. Lee, J. Lee, J. S. Kim, H. E. Lee, H. Yeon, H. S. Kum, S.-H. Bae, V. Bulovic, K. J. Yu, K. Lee, K. Chung, Y. J. Hong, A. Ougazzaden, J. Kim, Vertical full-colour micro-LEDs via 2D materials-based layer transfer. *Nature* **614**, 81–87 (2023).

6. H. Kum, D. Lee, W. Kong, H. Kim, Y. Park, Y. Kim, Y. Baek, S.-H. Bae, K. Lee, J. Kim, Epitaxial growth and layer-transfer techniques for heterogeneous integration of materials for electronic and photonic devices. *Nat. Electron.* **2**, 439–450 (2019).

7. I. Roh, S. H. Goh, Y. Meng, J. S. Kim, S. Han, Z. Xu, H. E. Lee, Y. Kim, S.-H. Bae, Applications of remote epitaxy and van der Waals epitaxy. *Nano Converg.* **10**, 20 (2023).

8. Y. Qu, Y. Xu, B. Cao, Y. Wang, J. Wang, L. Shi, K. Xu, Long-range orbital hybridization in remote epitaxy: The nucleation mechanism of GaN on different substrates via single-layer graphene. *ACS Appl. Mater. Interfaces* **14**, 2263–2274 (2022).

9. C. S. Chang, K. S. Kim, B. I. Park, J. Choi, H. Kim, J. Jeong, M. Barone, N. Parker, S. Lee, X. Zhang, K. Lu, J. M. Suh, J. Kim, D. Lee, N. M. Han, M. Moon, Y. S. Lee, D. H. Kim, D. G. Schlom, Y. J. Hong, J. Kim, Remote epitaxial interaction through graphene. *Sci. Adv.* **9**, eadj5379 (2023).

10. Y. Kim, J. Watt, X. Ma, T. Ahmed, S. Kim, K. Kang, T. S. Luk, Y. J. Hong, J. Yoo, Fabrication of a microcavity prepared by remote epitaxy over monolayer molybdenum disulfide. *ACS Nano* **16**, 2399–2406 (2022).

11. X. Wang, J. Choi, J. Yoo, Y. J. Hong, Unveiling the mechanism of remote epitaxy of crystalline semiconductors on 2D materials-coated substrates. *Nano Converg.* **10**, 40 (2023).

12. C. Liu, X. Li, Y. Wang, Z. Zheng, B. Wu, W. He, X. Dong, Z. Zhang, B. Chen, J. Huang, Z. An, C. Zheng, G. Huang, Y. Mei, Remote epitaxy and exfoliation of vanadium dioxide via sub-nanometer thick amorphous interlayer. *Nat. Commun.* **16**, 150 (2025).

13. Y. Song, D. Shoemaker, J. H. Leach, C. McGray, H.-L. Huang, A. Bhattacharyya, Y. Zhang, C. U. Gonzalez-Valle, T. Hess, S. Zhukovsky, K. Ferri, R. M. Lavelle, C. Perez, D. W. Snyder, J.-P. Maria, B. Ramos-Alvarado, X. Wang, S. Krishnamoorthy, J. Hwang, B. M. Foley, S. Choi, Ga_2O_3-on-SiC composite wafer for thermal management of ultrawide bandgap electronics. *ACS Appl. Mater. Interfaces* **13**, 40817–40829 (2021).

14. Z. Qu, Y. Xie, T. Zhao, W. Xu, Y. He, Y. Xu, H. Sun, T. You, G. Han, Y. Hao, X. Ou, Extremely low thermal resistance of β-Ga_2O_3 MOSFETs by co-integrated design of substrate engineering and device packaging. *ACS Appl. Mater. Interfaces* **16**, 57816–57823 (2024).

15. H. Takane, K. Kaneko, Y. Ota, S. Fujita, Initial nucleation scheme of Ga_2O_3 on (0001) sapphire by mist CVD for the growth of α-phase. *Jpn. J. Appl. Phys.* **60**, 055501 (2021).

16. E. Ahmadi, Y. Oshima, Materials issues and devices of α- and β-Ga_2O_3. *J. Appl. Phys.* **126**, 160901 (2019).

17. K. Kaneko, S. Fujita, T. Shinohe, K. Tanaka, Progress in α-Ga_2O_3 for practical device applications. *Jpn. J. Appl. Phys.* **62**, SF0803 (2023).

18. S. Y. Oh, Y. J. Jeong, I. Kang, J.-H. Park, M. J. Yeom, D.-W. Jeon, G. Yoo, A 2.8 kV breakdown voltage α-Ga_2O_3 MOSFET with hybrid Schottky drain contact. *Micromachines* **15**, 133 (2024).

19. L. Liu, T. Li, L. Ma, W. Li, S. Gao, W. Sun, R. Dong, X. Zou, D. Fan, L. Shao, C. Gu, N. Dai, Z. Yu, X. Chen, X. Tu, Y. Nie, P. Wang, J. Wang, Y. Shi, X. Wang, Uniform nucleation and epitaxy of bilayer molybdenum disulfide on sapphire. *Nature* **605**, 69–75 (2022).

20. L. Li, Q. Wang, F. Wu, Q. Xu, J. Tian, Z. Huang, Q. Wang, X. Zhao, Q. Zhang, Q. Fan, X. Li, Y. Peng, Y. Zhang, K. Ji, A. Zhi, H. Sun, M. Zhu, J. Zhu, N. Lu, Y. Lu, S. Wang, X. Bai, Y. Xu, W. Yang, N. Li, D. Shi, L. Xian, K. Liu, L. Du, G. Zhang, Epitaxy of wafer-scale single-crystal MoS_2 monolayer via buffer layer control. *Nat. Commun.* **15**, 1825 (2024).

21. G. R. Lee, C. Yang, R. B. K. Chung, Uniformity and thickness control of MoS_2 during thermolysis. *Appl. Sci. Converg. Technol.* **33**, 18–22 (2024).

22. H. Li, Q. Zhang, C. C. R. Yap, B. K. Tay, T. H. T. Edwin, A. Olivier, D. Baillargeat, From bulk to monolayer MoS_2: Evolution of Raman scattering. *Adv. Funct. Mater.* **22**, 1385–1390 (2012).

23. I. Irfan, S. Golovynskyi, M. Bosi, L. Seravalli, O. A. Yeshchenko, B. Xue, D. Dong, Y. Lin, R. Qiu, B. Li, J. Qu, Enhancement of Raman scattering and exciton/trion photoluminescence of monolayer and few-layer MoS_2 by by Ag nanoprisms and nanoparticles: Shape and size effects. *J. Phys. Chem. C* **125**, 4119–4132 (2021).

24. J. Pei, J. Yang, R. Xu, Y. H. Zeng, Y. W. Myint, S. Zhang, J. C. Zheng, Q. Qin, X. Wang, W. Jiang, Y. Lu, Exciton and trion dynamics in bilayer MoS_2. *Small* **11**, 6384–6390 (2015).

25. R. Luo, W. W. Xu, Y. Zhang, Z. Wang, X. Wang, Y. Gao, P. Liu, M. Chen, Van der Waals interfacial reconstruction in monolayer transition-metal dichalcogenides and gold heterojunctions. *Nat. Commun.* **11**, 1011 (2020).

26. Y. Oshima, K. Kawara, T. Oshima, M. Okigawa, T. Shinohe, Phase-controlled epitaxial lateral overgrowth of α-Ga_2O_3 by halide vapor phase epitaxy. *Jpn. J. Appl. Phys.* **59**, 025512 (2020).

27. Y. Choi, C. Kim, Y. Hwang, S. Park, H. Y. Kang, S. Chakraborty, R. B. K. Chung, Fluorine-doped n-type α-Ga_2O_3 and its phase stability. *Cryst. Growth Des.* **25**, 1023–1029 (2025).

28. T. C. Ma, X. H. Chen, Y. Kuang, L. Li, J. Li, F. Kremer, F.-F. Ren, S. L. Gu, R. Zhang, Y. D. Zheng, H. H. Tan, C. Jagadish, J. D. Ye, On the origin of dislocation generation and annihilation in α-Ga_2O_3 epilayers on sapphire. *Appl. Phys. Lett.* **115**, 182101 (2019).

29. R. Jinno, K. Kaneko, S. Fujita, Thermal stability of α-Ga_2O_3 films grown on c-plane sapphire substrates via mist-CVD. *AIP Adv.* **10**, 115013 (2020).

30. R. Mullen, J. W. Roberts, P. R. Chalker, R. A. Oliver, B. Hourahine, F. C. P. Massabuau, Atomic scale observation of threading dislocations in α-Ga_2O_3. *AIP Adv.* **14**, 115018 (2024).

31. S.-H. Bae, K. Lu, Y. Han, S. Kim, K. Qiao, C. Choi, Y. Nie, H. Kim, H. S. Kum, P. Chen, W. Kong, B.-S. Kang, C. Kim, J. Lee, Y. Baek, J. Shim, J. Park, M. Joo, D. A. Muller, K. Lee, J. Kim, Graphene-assisted spontaneous relaxation towards dislocation-free heteroepitaxy. *Nat. Nanotechnol.* **15**, 272–276 (2020).

32. S. Lee, J. Kim, B.-I. Park, H. I. Kim, C. Lim, E. Lee, J. Y. Yang, J. Choi, Y. J. Hong, C. S. Chang, H. S. Kum, J. Kim, K. Lee, H. Kim, G.-C. Yi, GaN remote epitaxy on a pristine graphene buffer layer via controlled graphitization of SiC. *Appl. Phys. Lett.* **125**, 252102 (2024).

33. W. Kong, H. Li, K. Qiao, Y. Kim, K. Lee, Y. Nie, D. Lee, T. Osadchy, R. J. Molnar, D. K. Gaskill, R. L. Myers-Ward, K. M. Daniels, Y. Zhang, S. Sundram, Y. Yu, S. Bae, S. Rajan, Y. Shao-Horn, K. Cho, A. Ougazzaden, J. C. Grossman, J. Kim, Polarity governs atomic interaction through two-dimensional materials. *Nat. Mater.* **17**, 999–1004 (2018).

34. H. Y. Kang, H. Kang, E. Lee, G. R. Lee, R. B. K. Chung, Sn-induced phase stabilization and enhanced thermal stability of κ-Ga_2O_3 grown by mist chemical vapor deposition. *ACS Omega* **6**, 31292–31298 (2021).

35. S. W. Bedell, P. Lauro, J. A. Ott, K. Fogel, D. K. Sadana, Layer transfer of bulk gallium nitride by controlled spalling. *J. Appl. Phys.* **122**, 025103 (2017).

36. H. Kim, J. C. Kim, Y. Jeong, J. Yu, K. Lu, D. Lee, N. Kim, H. Y. Jeong, J. Kim, S. Kim, Role of transferred graphene on atomic interaction of GaAs for remote epitaxy. *J. Appl. Phys.* **130**, 174901 (2021).

37. Y. Zang, S. Niu, Y. Wu, X. Zheng, J. Cai, J. Ye, Y. Xie, Y. Liu, J. Zhou, J. Zhu, X. Liu, G. Wang, Y. Qian, Tuning orbital orientation endows molybdenum disulfide with exceptional alkaline hydrogen evolution capability. *Nat. Commun.* **10**, 1217 (2019).

38. A. H. Castro Neto, F. Guinea, N. M. R. Peres, K. S. Novoselov, A. K. Geim, The electronic properties of graphene. *Rev. Mod. Phys.* **81**, 109–162 (2009).

39. A. K. Geim, K. S. Novoselov, The rise of graphene. *Nat. Mater.* **6**, 183–191 (2007).

40. J. A. Wilson, A. D. Yoffe, The transition metal dichalcogenides discussion and interpretation of the observed optical, electrical and structural properties. *Adv. Phys.* **18**, 193–335 (1969).

41. G.-B. Liu, D. Xiao, Y. Yao, X. Xu, W. Yao, Electronic structures and theoretical modelling of two-dimensional group-VIB transition metal dichalcogenides. *Chem. Soc. Rev.* **44**, 2643–2663 (2015).

42. K. Uno, M. Ohta, I. Tanaka, Growth mechanism of α-Ga_2O_3 on a sapphire substrate by mist chemical vapor deposition using acetylacetonated gallium source solutions. *Appl. Phys. Lett.* **117**, 052106 (2020).

43. K. H. Lee, C. S. Tan, Y. Wang, B. Wang, L. Zhang, W. A. Sasangka, S. C. Goh, S. Bao, K. E. Lee, E. A. Fitzgerald, Monolithic integration of Si-CMOS and III-V-on-Si through direct wafer bonding process. *IEEE J. Electron Devices Soc.* **6**, 571–578 (2018).

44. J. Jeong, S. K. Kim, J. Kim, D.-M. Geum, D. Kim, E. Jo, H. Jeong, J. Park, J.-H. Jang, S. Choi, I. Kwon, S. Kim, Heterogeneous and monolithic 3D integration of III–V-based radio frequency devices on Si CMOS circuits. *ACS Nano* **16**, 9031–9040 (2022).

45. Y. Son, B. Frost, Y. Zhao, R. L. Peterson, Monolithic integration of high-voltage thin-film electronics on low-voltage integrated circuits using a solution process. *Nat. Electron.* **2**, 540–548 (2019).

46. J. Yang, Y. Gu, E. Lee, H. Lee, S. H. Park, M.-H. Cho, Y. H. Kim, Y.-H. Kim, H. Kim, Wafer-scale synthesis of thickness-controllable MoS_2 films via solution-processing using a dimethylformamide/n-butylamine/2-aminoethanol solvent system. *Nanoscale* **7**, 9311–9319 (2015).

47. G. Kresse, J. Hafner, Ab initio molecular dynamics for liquid metals. *Phys. Rev. B* **47**, 558–561 (1993).

48. J. P. Perdew, K. Burke, M. Ernzerhof, Generalized Gradient Approximation Made Simple. *Phys. Rev. Lett.* **77**, 3865–3868 (1996).

49. S. Grimme, J. Antony, S. Ehrlich, H. Krieg, A consistent and accurate ab initio parametrization of density functional dispersion correction (DFT-D) for the 94 elements H-Pu. *J. Chem. Phys.* **132**, 154104 (2010).

50. D. Dardzinski, M. Yu, S. Moayedpour, N. Marom, Best practices for first-principles simulations of epitaxial inorganic interfaces. *J. Phys. Condens. Matter* **34**, 233002 (2022).

51. X. Zou, Y. Liu, B. I. Yakobson, Predicting dislocations and grain boundaries in two-dimensional metal-disulfides from the first principles. *Nano Lett.* **13**, 253–258 (2013).

52. G. Pilania, B. J. Thijsse, R. G. Hoagland, I. Lazić, S. M. Valone, X.-Y. Liu, Revisiting the Al/Al_2O_3 interface: Coherent interfaces and misfit accommodation. *Sci. Rep.* **4**, 4485 (2014).

53. E. A. Soares, M. A. Van Hove, C. F. Walters, K. F. McCarty, Structure of the α-Al_2O_3 (0001) surface from low-energy electron diffraction: Al termination and evidence for anomalously large thermal vibrations. *Phys. Rev. B* **65**, 195405 (2002).

54. T. Kurita, K. Uchida, A. Oshiyama, Atomic and electronic structures of α-Al_2O_3 surfaces. *Phys. Rev. B* **82**, 155319 (2010).

55. Y. Park, C. Ahn, J.-G. Ahn, J. H. Kim, J. Jung, J. Oh, S. Ryu, S. Kim, S. C. Kim, T. Kim, H. Lim, Critical role of surface termination of sapphire substrates in crystallographic epitaxial growth of MoS_2 using inorganic molecular precursors. *ACS Nano* **17**, 1196–1205 (2023).

56. J. E. Samaniego-Benitez, R. Mendoza-Cruz, L. Bazán-Díaz, A. Garcia-Garcia, M. J. Arellano-Jimenez, J. F. Perez-Robles, G. Plascencia-Villa, J. J. Velázquez-Salazar, E. Ortega, S. E. Favela-Camacho, M. José-Yacamán, Synthesis and structural characterization of MoS_2 micropyramids. *J. Mater. Sci.* **55**, 12203–12213 (2020).

57. H. Long, L. Chan, A. Harley-Trochimczyk, L. E. Luna, Z. Tang, T. Shi, A. Zettl, C. Carraro, M. A. Worsley, R. Maboudian, 3D MoS_2 aerogel for ultrasensitive NO_2 detection and its tunable sensing behavior. *Adv. Mater. Interfaces* **4**, 1700217 (2017).

58. K. M. Ponnusamy, N. Raveendran, S. Durairaj, S. K. Eswaran, S. Chandramohan, Spectroscopic visualization of intermediate phases during CVD synthesis of MoS_2. *J. Phys. Chem. Solid* **182**, 111575 (2023).

59. M. Fantauzzi, B. Elsener, D. Atzei, A. Rigoldi, A. Rossi, Exploiting XPS for the identification of sulfides and polysulfides. *RSC Adv.* **5**, 75953–75963 (2015).
